# Highly sensitive and label-free detection of SARS-CoV-2 proteins via surface plasmon resonance using biofunctionalization with 1 nm thick carbon nanomembranes

**DOI:** 10.1038/s41598-025-16342-5

**Published:** 2025-08-25

**Authors:** Ghazaleh Eshaghi, David Kaiser, Hamid Reza Rasouli, Rania Ennaciri, Martha Frey, Christof Neumann, Dominik Gary, Tobias Fischer, Katrin Frankenfeld, Andrey Turchanin

**Affiliations:** 1 Institute of Physical Chemistry, Friedrich Schiller University Jena, 07743 Jena, Germany; 2 fzmb GmbH, Forschungszentrum für Medizintechnik und Biotechnologie, 99947 Bad Langensalza, Germany; 3 Abbe Center of Photonics, Friedrich Schiller University Jena, 07745 Jena, Germany; 4 Jena Center for Soft Matter (JCSM), 07743 Jena, Germany

**Keywords:** Biosensors, SARS-CoV-2, Surface plasmon resonance, Two-dimensional materials, Carbon nanomembrane, Surface functionalization, Biotechnology, Nanoscience and technology

## Abstract

Here we report a novel platform for the detection of nucleocapsid (N) and receptor-binding domain (RBD) of spike (S) proteins of SARS-CoV-2 viruses using the surface plasmon resonance (SPR) technique. We demonstrate that the functionalization of SPR sensors with molecular 2D materials − 1 nm thick carbon nanomembranes (CNMs) significantly enhances sensitivity. CNMs terminated with azide linker (N_3_-CNM) enable covalent bonding of SARS-CoV-2 antibodies for specific immobilization of the N- and S-proteins to the sensor surface. The successful and stable hierarchical functionalization is confirmed by multiparametric SPR measurements complemented with X-ray photoelectron spectroscopy and polarization modulation infrared reflection absorption spectroscopy. The obtained equilibrium dissociation constants (*K*_*D*_) for the N-protein and the S-protein in the physiological buffer are 570 ± 50 pM and 22 ± 2 pM and the low detection limits (*LOD*s) are ~ 190 pM and ~ 10 pM, respectively. The high specificity of the developed sensors is shown via their negligible cross-reactivity with SARS-CoV-1 and MERS-CoV proteins. Finally, detection of SARS-CoV-2 proteins in nasopharyngeal swab samples with the *LOD* of ~ 40 pM is demonstrated. The proposed methodology enables the development of biosensors that cover clinically relevant range for the direct and immediate detection of SARS-CoV-2 without any amplification or labeling.

**Supplementary Information:**

The online version contains supplementary material available at 10.1038/s41598-025-16342-5.

## Introduction

Since the COVID-19 pandemic there has been a demand for rapid, early-stage and accurate detection of viral infections such as the severe acute respiratory syndrome coronavirus type 2 (SARS-CoV-2)^1,2^. Conventional diagnostic methods, such as reverse transcription polymerase chain reaction (RT-PCR), although highly sensitive, are time-consuming, require amplification steps, and depend on centralized laboratory infrastructure, which limits their speed and accessibility for point-of-care use^3^. Surface plasmon resonance (SPR) instruments are widely used for the scientific development of biosensors as they provide analysis of molecular interactions between viral targets and their specific biorecognition elements^4–6^. SPR-based sensing is a promising approach for the rapid detection of the SARS-CoV-2 virus, as it offers short response time and real-time monitoring of binding activities^7^. Furthermore, SPR analysis can be used to quantify the kinetics of binding interactions such as association and dissociation dynamics and binding affinity of SARS-CoV-2 antibody-antigen^8^. Various methods are currently applied to immobilize bioreceptor molecules on the SPR sensor chip. To this end, physical adsorption mechanisms (ion exchange, van der Waals interactions, hydrogen bonds) are highly unfavorable as they suffer from a lack of reproducibility of the binding, non-specificity of the detection and mass transport limitation of the analyte^9,10^. Surface functionalization engineering is a key to overcoming the mentioned drawbacks. A variety of different functionalization strategies^11,12^ have been implemented on SPR interfaces, leading to ongoing advancements in affinity-capture biosensor surfaces^13,14^. Among these, self-assembled monolayers (SAMs) are widely employed to immobilize biorecognition molecules on gold surfaces^12,15^. While SAM-based functionalization methods have proven effective, further improvements in stability and sensitivity can be achieved through the cross-linking of SAMs, which can enhance sensor performance in achieving a low limit of detection (*LOD*), particularly important for detecting pathogens like SARS-CoV-2.

The sensitivity of SPR sensors is highly dependent on the changes in the refractive index of the medium close to the sensor surface. Furthermore, the proximity of the bio-interaction to the sensor surface is crucial in SPR sensors due to the exponential decrease of the evanescent field generated during surface plasmon resonance^16^. Novel nanomaterials are being explored to achieve highly sensitive biosensors^17^. In particular, it has been widely shown that a two-dimensional (2D) material-metal hybrid structure can enhance the sensitivity and stability of SPR based biosensors^18,19^. However, in these approaches either the surface functional layers are too thick or the surface chemistry of these structures hinders versatile biofunctionalization resulting in a deficiency of immobilizing specific biorecognition elements. In addition, the refractive index of surface functionalization layers frequently matches that of biomolecules, largely due to their comparable high-water content, which in turn constrains the sensitivity of SPR sensors.

To address these issues, we employed carbon nanomembranes (CNMs)—molecularly thin, two-dimensional organic sheets derived by crosslinking of aromatic SAMs—which effectively enable the immobilization of capture molecules and have previously been used for the immobilization of polymers^20^, dyes^21^, His-tagged proteins^22^, and aptamers^23^. CNMs are two-dimensional sheets fabricated from organic SAMs^24^. The synthesis of CNMs is commonly achieved through the exposure of SAMs to low-energy electrons, which induces their crosslinking into the well-defined nanomembranes with specific area, thickness and surface functionality^25,26^. SAMs with nitro groups allow for controlled functionalization of the CNM after their conversion into amino groups^27^. In this work, we employ ~ 1 nm-thick azide-functionalized CNM (N_3_-CNM) as a novel 2D biorecognition platform for the detection of nucleocapsid protein (N-protein) and the receptor-binding domain (RBD) of the spike protein (S-protein), two targets of a great importance for the rapid diagnostic and monitoring of individuals with COVID-19, using the SPR technique.

The N-protein, with an estimated copy number of approximately 1000 per viral particle^28^, represents an abundant and stable target for early and sensitive detection of SARS-CoV-2 infection. The S-protein, although less abundant, mediates viral entry into host cells via ACE2 binding and serves as a key target for both diagnostics and therapeutic monitoring due to its central role in infectivity and the immune response^29–31^. Various surface blocking agents were examined and casein was found to be the most effective in reducing the non-specific adsorption of antigens. Each individual functionalization step was studied by the SPR technique in real-time under physiological conditions and further complemented by surface science characterization using X-ray photoelectron spectroscopy (XPS) in vacuum and polarization-modulation infrared reflection absorption spectroscopy (PM-IRRAS) at ambient pressure. We analyzed the binding kinetics, measured the equilibrium dissociation constant (*K*_*D*_) and investigated the adsorbed molecular mass of the biomolecules as well as the thickness changes upon binding using a multiparametric SPR, which involves simultaneous angular measurements at multiple wavelengths (670 nm, 785 nm, and 980 nm) to independently extract the refractive index and thickness of adsorbed layers. The N-protein antibody shows excellent specificity, evidenced by negligible cross-reactivity with other coronaviruses, including SARS-CoV-1 and MERS-CoV. Finally, highly sensitive detection of SARS-CoV-2 S-protein in nasopharyngeal swab samples was demonstrated.

## Results and discussion

**Fig. 1:**
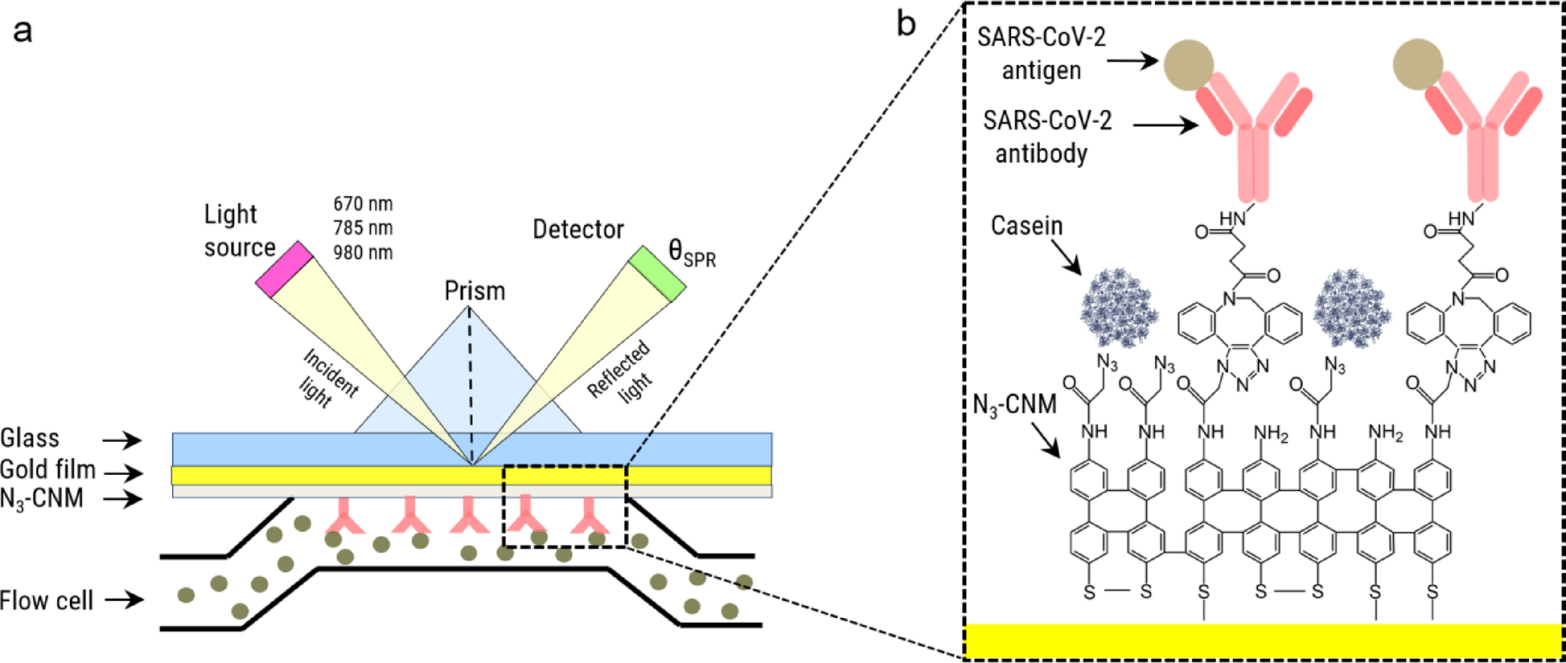
Schematic illustration of the basic SPR experimental setup for studying the specific binding of a SARS-CoV-2 protein to its complementary antibody. (**a**) Multiparametric SPR setup in the Kretschmann configuration^35^. (**b**) Enlarged view of the bio-functionalized surface of the SPR chip including the target proteins.

Figure 1a presents a schematic of the multiparametric SPR system. This system operates at three wavelengths, thereby enhancing measurement accuracy and sensitivity by simultaneously quantifying both the thickness and the refractive index of the analyte layer, which facilitates precise characterization of complex samples with variable optical properties. We monitored real-time shift of the resonance angle of surface plasmons, *θ*_*SPR*_, with three different laser wavelengths (670 nm, 785 nm and 980 nm) upon injection of various solutions into the flow cell. During the measurements, the binding response in two channels, signal and reference channels, was recorded in parallel. A biofunctionalization scheme of the signal channel is presented in Fig. 1b. Initially, a 4’-nitro-[1,1’]-biphenyl-4-thiol (NBPT) SAM is formed on a gold-coated SPR sensor and afterward converted into an ~ 1 nm thick amino-terminated CNM (NH_2_-CNM) via low-energy electron irradiation^32^. Next, azidoacetyl chloride linker is grafted to the amino-groups forming an azide-terminated CNM (N_3_-CNM)^33^. Subsequently, SARS-CoV-2 antibodies are functionalized with dibenzocyclooctyne (DBCO) linkers using the N-hydroxysuccinimide (NHS) ester reaction^34^. These DBCO-modified antibodies are then covalently attached to azide-functionalized surfaces in the signal channel via copper-free click chemistry. Finally, the signal channel and the reference channel are passivated with a blocking agent (e.g., casein) to reduce non-specific protein adsorption. The target biomolecule solutions are introduced into both the signal channel, where antibodies were immobilized, and the reference channel, which served as a control measurement of the binding on a surface without antibodies. The functionalized SPR sensor demonstrates excellent repeatability and reproducibility, ensuring consistent performance across multiple measurements. Additionally, it exhibits outstanding storage stability, retaining its functionality for over a year when stored at 4 °C.

**Fig. 2:**
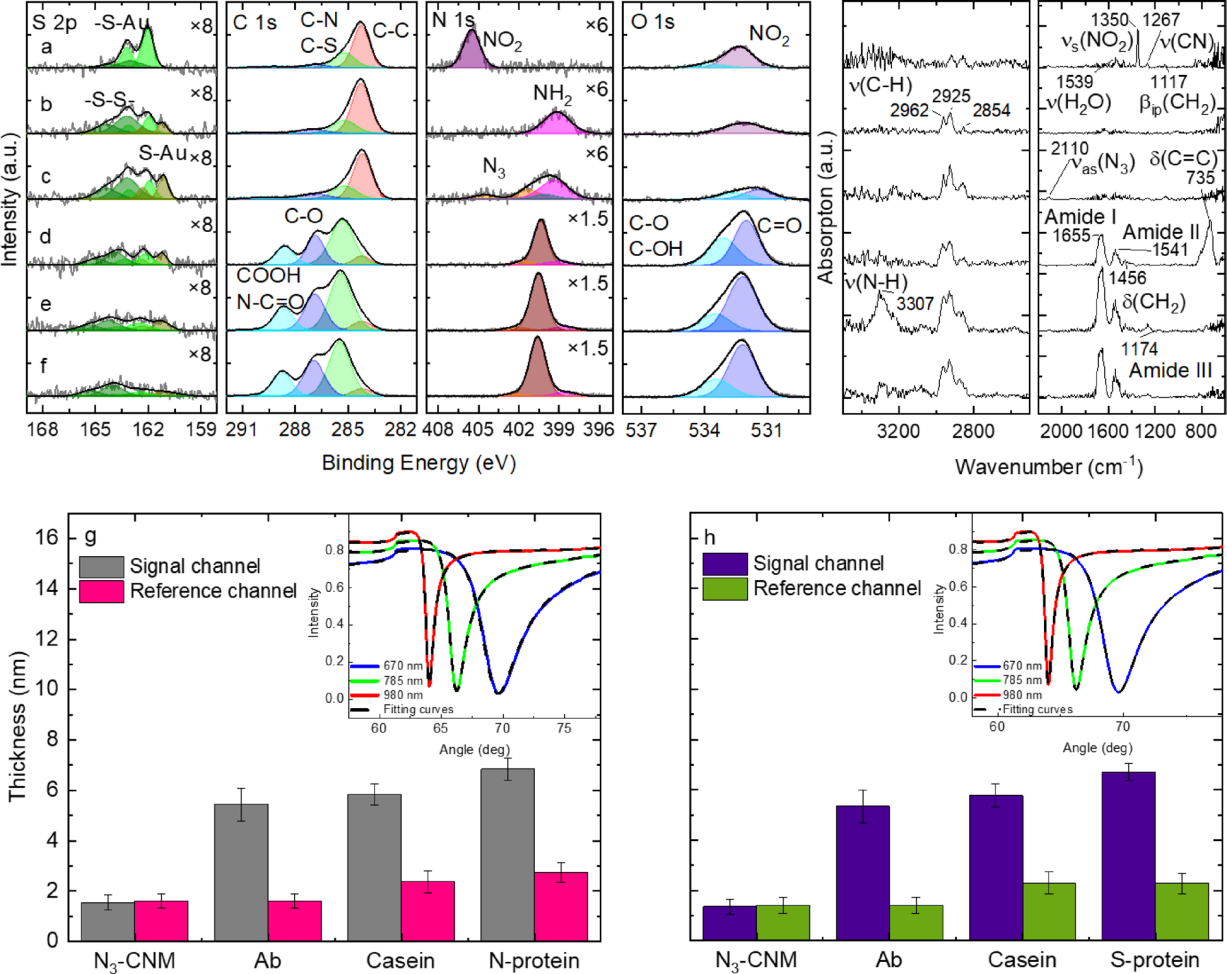
Subsequent XPS and IRRAS characterization of synthesis, chemical-functionalization bio-functionalization of the CNM. XP and IRRA spectra showing the S 2p, C 1s, N 1s, and O 1s peaks of six samples: (**a**) NBPT SAM, (**b**) NH_2_-CNM, (**c**) N_3_-CNM, (**d**) immobilized antibody on N_3_-CNM, (**e**) antibody-CNM functionalized with casein and (**f**) N-protein binding. The S 2p and N 1s spectra are multiplied by the given factors for a better representation of the spectra. Subsequent thickness measurements by multiparametric SPR for N-protein (**g**) and (**h**) S-protein assemblies. The inset shows the full SPR curves recorded upon the injection of the DBCO labelled anti-SARS-CoV-2 N-protein and S-protein antibody at three wavelengths (670 nm: blue line, 785 nm: red line and 980 nm: green line). Dashed black lines represent model calculations of the SPR curves.

To demonstrate the successful biofunctionalization of the SPR sensors, we performed detailed XPS and PM-IRRAS analyses of each functionalization step on the gold SPR sensor chips and gold on a silicon wafer, respectively. Figure 2a shows the high-resolution XP spectra of S 2p, C 1s, N 1s and O 1s of the formed NBPT SAM, which confirm its successful preparation^27^. After the electron irradiation of the NBPT SAM, the NH_2_-CNM is formed as can be followed from the conversion from the nitro (binding energy (BE) = 405.5 eV, purple) to amino (399.2 eV, magenta) groups visible in the N 1s spectrum (Fig. 2b). Next, the chemical functionalization of the CNM with azide groups is confirmed by the appearance of the new peaks in the N 1s spectrum (Fig. 2c, orange)^36^. Subsequently, the N-protein antibodies are covalently attached to the N_3_-CNM, leading to significant changes in the XP spectra (Fig. 2d). By successful immobilization of the antibody, the N 1s species at a BE of 404.5 eV (orange) assigned to the central nitrogen of the azide linker are vanishing, and an intense peak at a BE of 400.2 eV (brown), according to amino acids, is appearing. In the C 1s spectrum, intense peaks assigned to C-N bonds (285.3 eV, green), C-O/C = O bonds (286.8 eV, dark blue), and COOH/N-C = O bonds (288.6 eV, light blue) are visible. This leads to an increase of the effective thickness from 1.4 ± 0.2 nm for the N_3_-CNM to 3.5 ± 0.5 nm for the antibody-CNM. In the next step, the surface is passivated with the casein molecules. As casein consists of similar chemical groups as the antibody (C-C, C-N, C = O, N-C = O), no new features appear in the XP spectra; however, a clear increase in the effective thickness to 4.9 ± 0.7 nm is observed (Fig. 2e). Finally, the SARS-CoV-2 N-protein with the concentration of 8 nM is captured on the surface, leading to a further increase in the signal intensity as seen by the thickness increase to 5.7 ± 0.8 nm (Fig. 2f). For a more detailed XPS analysis, see Supporting Information (SI) Section 2.

The complementary PM-IRRAS measurements are also presented in Fig. 2. For the NBPT SAM (a) a strong band at 1345 cm^−1^ is observed, which we assign to the symmetric stretching mode ν_s_(-NO_2_)^28^. Furthermore, the ν(CH) stretching modes appear at 2922 cm^−1^ and 2858 cm^−1^ bands^27,37^. After electron irradiation, (b), all -NO_2_ vibrations disappear in the spectra as the nitro group is converted into an amine group. Next, the CNM is functionalized with azide groups to form an N_3_-CNM. In spectrum (c), the successful functionalization is confirmed by the appearance of the characteristic stretching vibration of azide groups at a wavenumber of 2110 cm^−1^^38^. After immobilization of the antibody on the N_3_-CNM surface, the spectra undergo a pronounced change (see Fig. 2d), which can be attributed to the multitude of vibrational characteristics exhibited in the monoclonal antibody. In particular, the amide bonds between amino acids in proteins are expected to lead to characteristic features, which are indeed visible at wavenumbers of 1653 cm^−1^, 1541 cm^−1^ and 1265 cm^−1^^39^. Thus, the PM-IRRAS results confirm the successful immobilization of the antibody, which is in agreement with the results obtained by XPS. Upon binding of casein, a new signal at 3305 cm^−1^ emerges corresponding to the NH stretching vibrations characteristic for casein^40^, further confirming the successful attachment of casein onto the surface. Finally, upon binding of the N-protein with a concentration of 8 nM on the surface, the distinctive features of Amide I, Amide II, and Amide III persist, signifying the presence of protein structures.

In the next step, the multiparametric SPR measurements were used to study the subsequent functionalization steps of the sensor surface in buffer solution, Fig. 2g and h (see SI Section 3 for details). Thus, for the N_3_-CNM we determine a thickness of 1.48 ± 0.12 nm; after attachment of N- and S-protein antibodies the thickness increased to 5.4 ± 0.5 nm and 5.3 ± 0.6 nm, respectively. The passivation with casein results in a further increase to 5.8 ± 0.2 nm and to 5.8 ± 0.4, for the CNMs functionalized with N- and S-protein antibodies, respectively. Finally, the additional presence of the target proteins led to a thickness increase by 0.99 ± 0.03 nm for the N-protein with a concentration of 8 nM and 0.93 ± 0.03 nm for the S-protein with a concentration of 1.6 nM, demonstrating that these proteins are attached to the antibody-functionalized surface. A slight increase of the thickness by 0.36 ± 0.03 nm was observed after injection of the N-protein in the reference channel which indicates some non-specific adsorption; for the S-protein such a change was not observed showing that the non-specific adsorption is negligible. A comparative analysis of the CNM thickness values obtained by XPS and SPR (see Table S1) shows their similar values before functionalization with biomolecules, whereas the thickness of the biofunctionalized CNMs obtained by XPS in vacuum is systematically lower in comparison to the values obtained by SPR in buffer solution. These results suggest that N_3_-CNM does not experience any significant swelling in water; on the other hand, the biofunctionalization layer loses some incorporated water molecules upon its introduction in vacuum^41^.

**Fig. 3:**
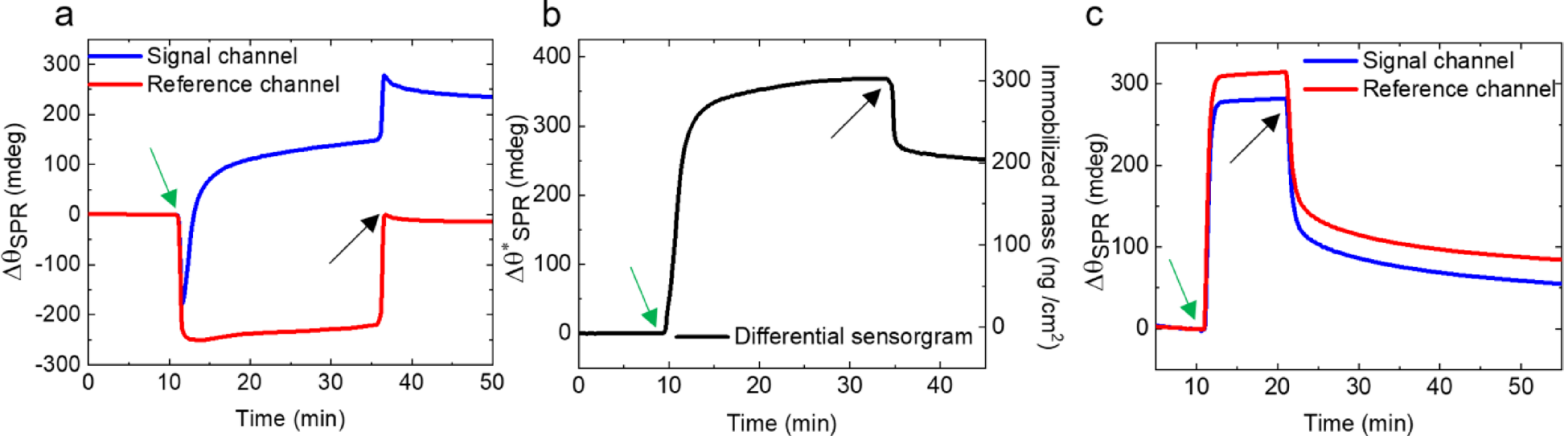
Biofunctionalization of N_3_-CNM with SARS-CoV-2 N-protein antibody and casein molecules. (**a**) Real-time surface plasmon resonance angle shift ($\:{\varDelta\:\theta\:}_{SPR}$) during the N-protein antibody immobilization (25 min) for the signal (blue curve) and the reference (red curve) channels. (**b**) Differential sensorgram ($\:{\varDelta\:\theta\:}_{SPR}^{*}$) during the N-protein antibody immobilization. (**c**) Time dependent $\:{\varDelta\:\theta\:}_{SPR}$ changes during the surface passivation with casein of the signal (blue curve) and the reference (red curve) channels. The start and the end of the injections are shown with green and black arrows, respectively.

Next, we present results of the in situ biofunctionalization of the N_3_-CNMs on the SPR sensors with monoclonal SARS-CoV-2 N-protein antibody obtained at a wavelength of 670 nm (for SARS-CoV-2 S-protein antibody see Figure S1 in the SI). Figure 3a shows the SPR sensorgrams representing the time dependent resonance angle shift, $\:{\varDelta\:\theta\:}_{SPR}$, which are calibrated to zero with respect to the initial values obtained in the physiological PBS-P buffer for both signal and reference channels. An injection of the acetate buffer with the antibody solution into the signal channel and the pure acetate buffer into the reference channel results in the respective a sharp increase/decrease of $\:{\varDelta\:\theta\:}_{SPR}$. During the N-protein antibody injection the $\:{\varDelta\:\theta\:}_{SPR}$ in the signal channel is increasing until saturation is reached, while it remains nearly constant in the reference channel. Figure 3b shows a differential sensorgram ($\:{\varDelta\:\theta\:}_{SPR}^{*}$) for the immobilization of N-protein antibody, obtained by subtraction of the $\:{\varDelta\:\theta\:}_{SPR}$ of the reference channel and the signal channels, which eliminates the bulk effects. As extracted from these data, an effective binding response, $\:{R}_{Ab}$, for the N-protein antibody is 250 ± 24 mdeg corresponding to the immobilized mass of 208 ± 21 ng/cm^2^ (see SI Section 5 for deteils). For the S-protein antibody, we obtained an effective binding response of 309 ± 32 mdeg corresponding to the immobilized mass of 257 ± 29 ng/cm^2^. We include these results in our estimation of the surface coverage for the N-protein and the S-protein antibodies on the SPR sensor surface of 26 ± 7% and 29 ± 8%, respectively (see SI Section 5).

Non-specific protein adsorption on the surface of SPR-based sensors reduces their sensitivity for detection of the target biomolecules and the use of blocking agents can be employed to reduce this impact^42,43^. To this end, we tested polyethylene glycol (PEG), bovine serum albumin (BSA), and casein^44,45^ for the surface passivation after immobilization of the N-protein antibody. We found out that particularly the passivation with casein improves equilibrium dissociation constant, sensitivity and signal-to-noise ratio (for details, see SI Sections 6 and 7). In Fig. 3c, the sensorgram curves illustrate the response following the introduction of casein, which continues until saturation occurs, indicating complete surface coverage of the areas lacking antibodies in the signal channel and of the complete surface in the reference channel. As a result, the binding response of the blocking agents in the reference channel is higher (92 ± 21 mdeg) than that in the signal channel (63 ± 18 mdeg). Thus, the binding response in the signal channel is reduced by 32 ± 10% compared to the reference channel, which correlates well with our estimate that 26 ± 7% of the surface are covered by N-protein antibodies. At the end of the casein injection, a drift is observed in both sensorgrams which is most probably due to desorption of some weakly bonded casein species from the sensor surface. After ~ 30 min the baselines of both sensorgrams become almost stable.

**Fig. 4:**
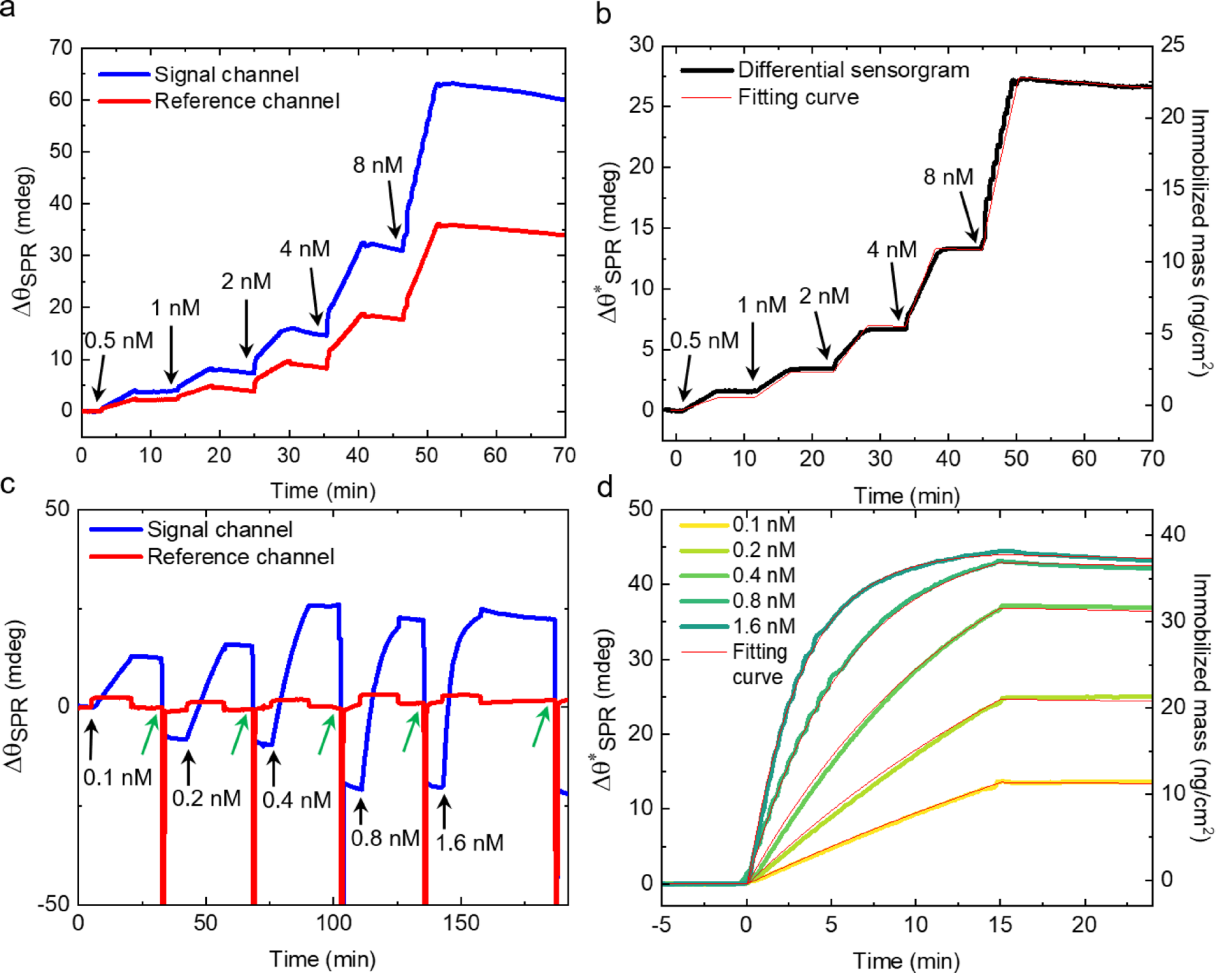
SPR-based detection of SARS-CoV-2 N-protein and S-protein using antibody-functionalized N_3_-CNM in the spiked samples prepared in the buffer solution. (**a**) Real-time resonance angle shift ($\:{\varDelta\:\theta\:}_{SPR}$) during the series of N-protein concentration injections (5 min for each concentration) into the signal (blue curve) and the reference (red curve) channels. (**b**) Differential sensorgram ($\:{\varDelta\:\theta\:}_{SPR}^{*}$) during the series of N-protein concentration injections (black curve) with the result of single-cycle kinetics fitting (red curve). (**c**) Real-time $\:{\varDelta\:\theta\:}_{SPR}$ during the series of S-protein concentration injections (15 min for each concentration) into the signal (blue curve) and the reference (red curve) channels. The green arrows indicate the time of the regeneration cycle. The black arrows in a, b, and c indicate the injection time for each concentration value. (**d**) The results of multi-cycle kinetics fitting (red curves) for differential responses ($\:{\varDelta\:\theta\:}_{SPR}^{*}$) of each S-protein concentration (see Table S6 in SI for the results of the calculation). The start of the protein injections and regeneration are shown with green and black arrows, respectively.

Next, we demonstrate the ability of the engineered SPR sensors to specifically detect N- and S-proteins of SARS-CoV-2 in the spiked samples prepared in the buffer solution. First, we describe results for the N-protein. Figure 4a shows sensorgrams of the signal and reference channels during the injection of a series of N-protein concentrations. Upon injection of a 500 pM solution of N-protein, a clear difference between the signal and reference channel sensorgrams is observed. In Fig. 4b the differential sensorgram of the signal and reference channel is presented, which enables minimizing the effect of the non-specific adsorption in the former. The differential sensorgram shows an incremental increase of the binding response of N-protein with increasing concentration. Note that complete regeneration of sensor surface from the bound N-proteins was not possible (see SI Section 8 for details). Following the injection of an 8 nM concentration, the surface binding response approaches saturation. Upon further injection at 16 nM, no significant increase in the binding response is observed, indicating that the surface is nearly fully saturated (see SI Section 9, Figure S5b for details). The obtained results were analyzed quantitatively by applying the Langmuir model^46^. We found out that the N-protein binds to its corresponding antibody with an association rate constant (*k*_*a*_) of 300 ± 18 × 10^4^ M^−1^s^−1^ and forms a stable complex with a dissociation rate constant (*k*_*d*_) of 170 ± 10 × 10^− 5^ s^−1^, which results in an effective equilibrium dissociation constant (*K*_*D*_*)* of 570 ± 50 pM (see SI Section 7 and Table S5 for details). It is important to note that while mass transport limitation can influence surface-based kinetic measurements in general, several experimental observations suggest that it is not dominant in our system, since in our experiments we observed strongly concentration-dependent curvature of the binding response during the association phase as well as a minimal signal decay during an extended dissociation phase of 30 min (see Figure S4) supporting a stable antigen–antibody interaction. The obtained *K*_*D*_ value for the binding of SARS-CoV-2 N-protein with the N-protein antibody immobilized on CNM is more than one order of magnitude lower than the affinity constant of comparable antibodies immobilized on the nitrocellulose (NC) membrane using lateral flow immunoassay (LFIA)-based biosensor for COVID-19^47^. The smallest N-protein concentration that could be clearly detected was 250 pM. We calculated the *LOD* defined as the analyte concentration that generates an SPR signal three times greater than the noise^48^ for the measurements with casein as a blocking agent, was ~ 190 pM (see SI Section 1.1 and SI Table S5). The binding response for the N-protein at the end of the last sample injection was 27 ± 2 mdeg, which corresponds to an immobilized mass of 23 ± 2 ng/cm² and a relative surface coverage of 27 ± 12% of the available specific binding sites, see SI Section 5.

Figure 4c presents results for the S-protein detection for five different concentrations in a range from 0.1 nM to 1.6 nM. In contrast to the N-protein, the sensor surface could be regenerated after each injection enabling removal of the bound antigens. A gradual baseline shift is observed in the signal channel during the initial regeneration cycles, which likely results from the removal of loosely adsorbed or non-specifically bound antibodies that are not covalently attached. After approximately three regeneration cycles, the baseline stabilizes, indicating that only stably immobilized antibodies remain, covalently linked via the DBCO–azide click chemistry. While some antibodies were lost from the surface during each regeneration cycle, the overall functionality of the remaining antibodies was preserved. After up to five regeneration cycles, the maximum binding response (*R*_*max*_) remained at 75% of its initial value, indicating minimal impact on binding performance (see Table S5 in the SI for details). In comparison, the reference channel—blocked only with casein—shows minimal signal change, suggesting that casein desorption is not the primary cause of the observed signal decrease. In Fig. 4d, the differential sensorgram is presented (for the differential sensorgram of lower concentrations of S-protein see SI Section 9). The results show a stepwise increase in the binding response as the concentration of S-protein increases. At the highest studied concentration of 1.6 nM the binding response reaches the saturation level. A summary of the *k*_*a*_, *k*_*d*_, and *K*_*D*_ values derived from the Langmuir model (red curves) is shown in Table S6 of the SI. The high *k*_*a*_ of 200 ± 14 × 10^4^ M^−1^s^−1^ demonstrates a rapid initial antibody-antigen interaction, leading to a stable molecular complex, while the low *k*_*d*_ of 4.5 ± 0.3 × 10^− 5^ s^−1^ shows a strong binding affinity yielding a *K*_*D*_ value of 22 ± 2 pM. The *K*_*D*_ value for the binding of SARS-CoV-2 S-protein to its corresponding antibody is over 30 times lower than that for the N-protein. In addition, it is 2 to 4 orders of magnitude lower than the previously reported data^49,50^. The *LOD* for the S-protein at the end of the last sample injection is estimated to be only ~ 10 pM, see SI Table S6. The binding response of S-protein is about 44 ± 3 mdeg corresponding to an immobilized mass of 38 ± 3 ng/cm² and a relative surface coverage of 35 ± 9% of the available specific binding sites (see SI Section 5).

**Fig. 5:**
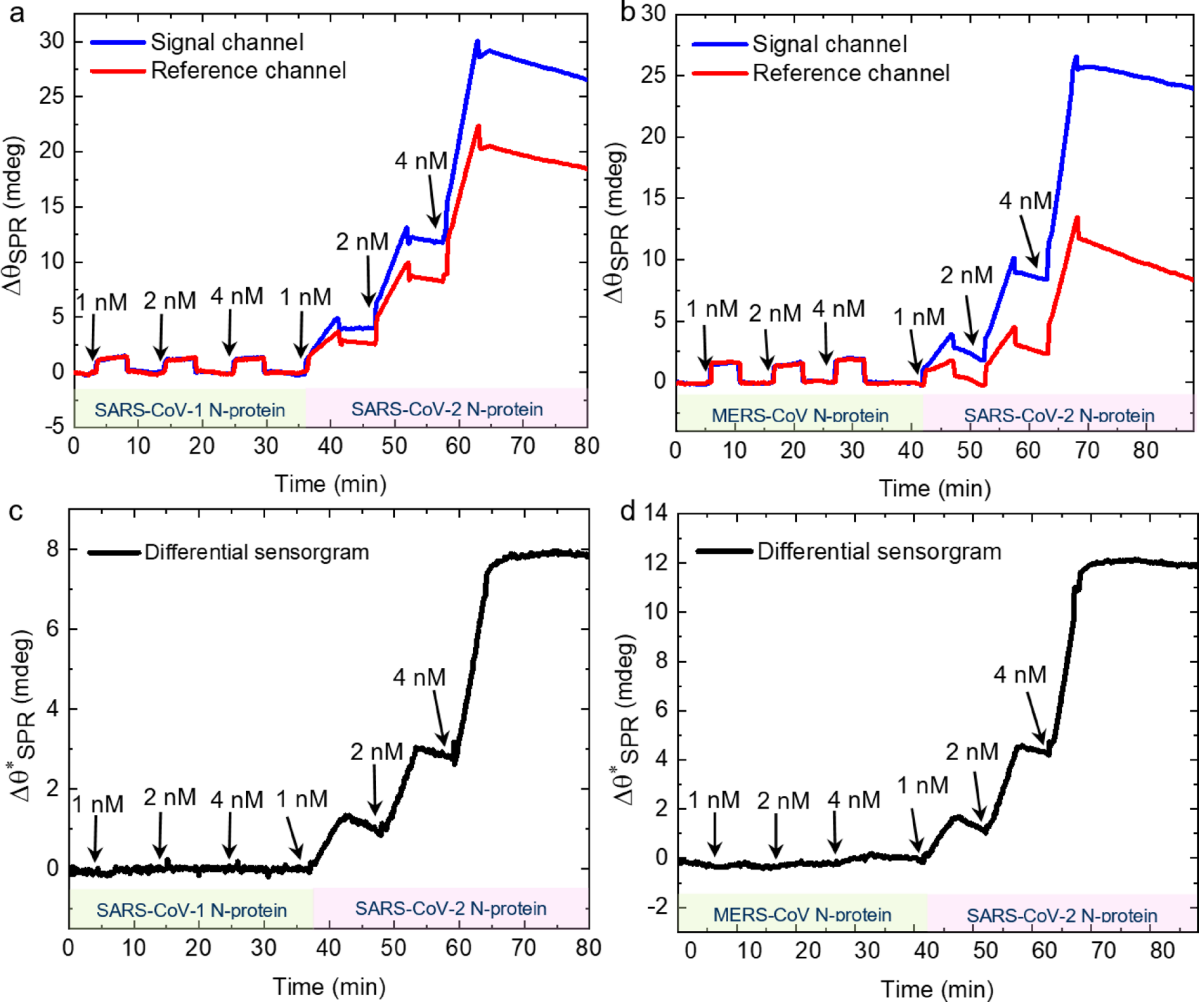
Specific detection of SARS-CoV-2 N-proteins in solutions containing SARS-CoV-1 and MERS-CoV N-proteins. (**a**, **b**) Real-time $\:{\varDelta\:\theta\:}_{SPR}$ response during a series of SARS-CoV-1 and MERS-CoV N-protein concentration injections (each concentration for 5 min) followed by the injections of the same-range concentrations of SARS-CoV-2 N-protein into the signal (blue curve) and the reference (red curve) channels, respectively. (**c**,** d**) Differential sensorgrams ($\:{\varDelta\:\theta\:}_{SPR}^{*}$) of a and b, respectively. The black arrows in each figure indicate the injection time for each concentration value. The start of the proteins injections is shown with black arrows.

After demonstrating a highly sensitive SPR-based detection of SARS-CoV-2 N-protein and S-protein antigens using biofunctionalization of N_3_-CNM, we demonstrate next a high selectivity of the developed sensor platform. Coronaviruses are single-stranded RNA viruses with a genome of approximately 30 kbp, which encode four structural proteins such as spike, envelope, matrix and nucleocapsid^51,52^. The N-protein has a more conserved structure and higher evolutionary similarity within the coronavirus family compared to the S-protein. Thus, N-protein antibodies can potentially demonstrate more cross-reactivity among the endemic coronaviruses^53^. Accordingly, we investigate the cross-reactivity of SARS-CoV-2 N-protein with SARS-CoV-1 and MERS-CoV N-proteins, which is essential for the specific diagnosis of COVID-19. Figure 5a and b show the sensorgrams of the signal (antibody/casein-CNM) and the reference (casein-CNM) channels upon the injection of SARS-CoV-1 N-protein and MERS-CoV N-protein, respectively, at concentrations of 1, 2 and 4 nM, followed by the injection of the same concentrations of SARS-CoV-2 N-protein. During the injection of SARS-CoV-1 and MERS-CoV N-proteins the response in the signal and reference channels are similar. Furthermore, once the injections are finished, the baseline returns to its original value suggesting that no binding takes place. After these measurements, when the same concentrations of SARS-CoV-2 N-protein are injected, a larger response of $\:{\varDelta\:\theta\:}_{SPR}$ is observed in the signal channel compared to the reference channel. Figure 5c and d show the differential sensorgrams of the results presented in Fig. 5a and b, respectively. During the injections of SARS-CoV-1 and MERS-CoV N-proteins, the sensorgram becomes a straight line with a mean value of zero, indicating that no binding occurred. However, immediately after the injection of SARS-CoV-2 N-protein, a clear binding response is observed. This pronounced difference demonstrates high specificity of the N-protein antibody towards SARS-CoV-2 N-protein.

**Fig. 6:**
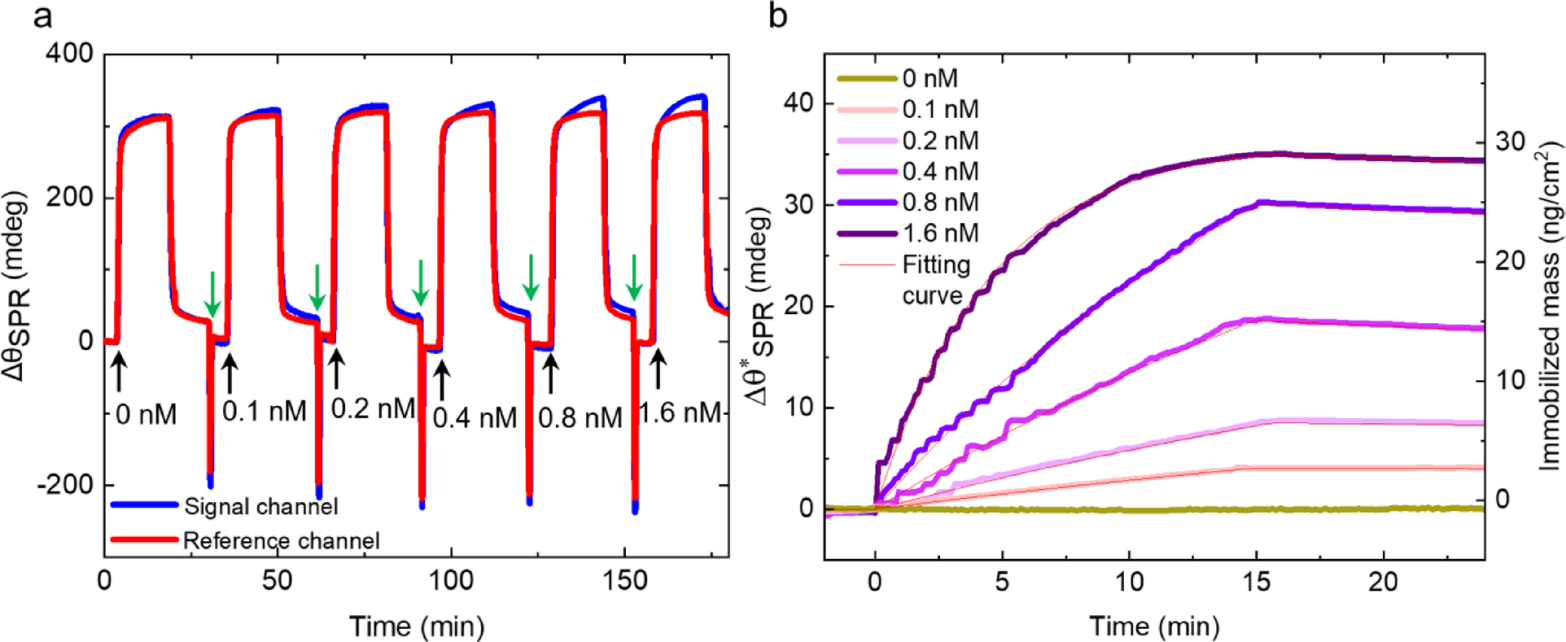
SPR-based detection of SARS-CoV-2 S-protein in spiked nasopharyngeal swab sample using antibody-functionalized N_3_-CNM. (**a**) Real-time $\:{\varDelta\:\theta\:}_{SPR}$ during the series of S-protein concentration injections (15 min for each concentration) into the signal (blue curve) and the reference (red curve) channels. The green arrows indicate the time of the regeneration cycle. The black arrows indicate the injection time for each concentration value. (**b**) The results of multi-cycle kinetics fitting (red curves) for differential responses ($\:{\varDelta\:\theta\:}_{SPR}^{*}$) of each S-protein concentration. The start of the injections and regeneration are shown with green and black arrows, respectively.

Finally, we evaluated the performance of the biofunctionalized N_3_-CNM in terms of the sensitivity and specificity for detecting SARS-CoV-2 antigens in complex biological media that is nasopharyngeal swab samples prepared in a lysate buffer. Figure 6a shows the sensorgrams of the signal (antibody-immobilized and casein-functionalized N_3_-CNM) and the reference (casein-functionalized N_3_-CNM) channels during the injection of a series of S-protein in the nasopharyngeal swab sample (negative sample) and the spiked nasopharyngeal swab samples (positive sample). After each concentration injection, the surface is regenerated. The signal channel’s specific and non-specific binding response to the S-protein is shown as the blue curve, while the red curve shows the signal generated only from the non-specific binding of the S-protein to the sensor surface. A clear difference between the responses of the signal and reference channels becomes visible after the injection of the sample spiked with 0.1 nM of the antigen. Figure 6b shows the differential sensorgrams of the data presented in Fig. 6a, including the model fits. As shown in Fig. 6b, the differential response to the positive samples is significantly higher than in the negative sample. The obtained *k*_*a*_ (52 ± 4 × 10^4^ M^−1^s^−1^) and *k*_*d*_ (5.7 ± 0.4 × 10^− 5^ s^−1^) values result in the binding of the antibody to the antigen with the *K*_*D*_ value of 109 ± 10 pM in spiked nasopharyngeal swab sample, see SI Table S7. Compared to measurements in physiological PBS-P buffer, both the *K*_*D*_ and *LOD* values showed a moderate increase when evaluated in nasopharyngeal swab matrix, with *K*_*D*_ rising from 22 pM to 109 pM (∼5-fold) and *LOD* increasing from ∼10 pM to ∼40 pM (∼4-fold). This behavior is consistent with known effects of complex biological media on SPR-based biosensing. Components present in clinical samples such as mucins, proteases, glycoproteins, salts, and enzymes can interfere with antigen–antibody binding by inducing steric hindrance, altering the local refractive index, or contributing to non-specific interactions at the sensor interface. Additionally, factors such as pH variability and ionic strength can modulate binding kinetics and reduce apparent affinity. These matrix-induced shifts in apparent affinity and sensitivity are widely reported and underscore the importance of assessing biosensor performance in clinically relevant environments^54^.

In order to assess the suitability of the developed biosensor for detecting the S-protein in clinical samples, we estimated the expected concentration range based on reported viral loads in nasopharyngeal swabs. Taking into account typical viral RNA copy numbers reported in clinical studies (~ 10^8.3^–10^10.5^ copies/ml), an extraction volume of 1 ml as typically used in rapid antigen tests, and the estimated number of spike proteins per virion (~ 150), the corresponding S-protein concentration was calculated to fall within the range of approximately 0.05 to 7.8 nM^50,55–58^. This range is clinically relevant for detecting SARS-CoV-2 antigens in patient samples using rapid diagnostic platforms (see SI Section 11 for the detailed calculation methodology). Given that our SPR biosensor has a *LOD* of approximately 40 pM (see SI Table S7), it is capable of detecting the clinically relevant concentrations of S-protein present in COVID-19 patient samples providing a higher sensitivity than previously reported approaches, see SI Section 11^59,60^.

## Conclusions

In this study we demonstrate the significant potential of N_3_-CNM as a robust and versatile platform for the biofunctionalization of SPR sensors. Specifically, we were targeting the detection of SARS-CoV-2 nucleocapsid and spike proteins. The use of 1 nm thick N_3_-CNM on a gold-coated SPR sensor chip enables the covalent attachment of SARS-CoV-2 antibodies in close proximity to the sensor surface enhancing the sensitivity and therewith precision of the detection. The sensor’s high sensitivity is evidenced by its remarkably low detection limits of *~* 190 pM for the N-protein and *~* 10 pM for the S-protein in physiological PBS-P buffer with the dissociation constant (*K*_*D*_) of 570 ± 50 pM and 22 ± 2 pM, respectively. The sensor’s specificity is further demonstrated by a negligible cross-reactivity with nucleocapsid proteins from SARS-CoV-1 and MERS-CoV. Furthermore, the successful detection of SARS-CoV-2 S-protein in nasopharyngeal swab samples, with a detection limit as low as *~* 40 pM, highlights practical applicability of the sensor. Our findings suggest that the N_3_-CNM offers a promising capability for surface biofunctionalization for highly sensitive and selective SARS-CoV-2 protein detection, which could be further integrated with more advanced biosensors without compromising its biofunctionality. The remarkable sensitivity and specificity presented by biofunctionalized CNMs pave the way for refining diagnostic and monitoring techniques for infectious diseases beyond COVID-19.

## Methods

### Materials

4’-nitro-[1,1’]-biphenyl-4-thiol (NBPT) was acquired from Taros Chemicals GmbH. N, N-dimethylformamide, extra dry 99.8%, AcroSeal™ acquired from VWR International GmbH. Azidoacetyl chloride, a 30% solution in ether, was acquired from SelectLab Chemicals GmbH. N, N-diisopropylethylamine, redistilled, was acquired from Sigma-Aldrich Chemie GmbH. Dibenzocyclooctyne (DBCO) labeled monoclonal SARS-CoV-2 nucleocapsid protein antibody (N-protein antibody), monoclonal SARS-CoV-2 spike protein antibody (S-protein antibody), S-protein (wild-type strain) receptor binding domain (RBD S-protein) and lysate buffer solution (EDTA (5–50 mM), detergent (0.2% v/v), protein (1% w/v), polymer (0.25% v/v) were acquired from fzmb GmbH, Forschungszentrum für Medizintechnik und Biotechnologie. SARS-CoV-2 N-protein (wild-type strain) was acquired from Virion\Serion GmbH. Casein buffer (#CBC1), 5,5% (w/v) biotin-free was acquired from Stereospecific Detection Technologies (SDT GmbH). Dibenzocyclooctyne (DBCO) labeled polyethylene glycol (DBCO-PEG) 20 kDa and 5 kDa were acquired from Vector Laboratories GmbH and Jena Bioscience GmbH, respectively. Phosphate buffered saline (PBS-P buffer 10x, aqueous buffer containing 0.2 M phosphate buffer with 27 mM KCl, 1.37 M NaCl and 0.5% Surfactant P20 (Tween 20)), glycine/HCl 10 mM pH = 2 and Immobilization buffer (10 mM sodium acetate pH 4.5) were acquired from Cytiva GmbH.

### Preparation of NH_2_-CNM

To prepare the NH_2_-CNMs, NBPT SAMs were formed on an oxygen plasma cleaned, ~ 50 nm polycrystalline gold-coated SPR sensor chip (from Bionavis Ltd., Tampere, Finland)^32^. The NBPT SAM is afterward irradiated by low-energy electrons (50 eV / 50 mC/cm^2^) in high vacuum (< 5 × 10^− 7^ mbar)^32^. The irradiation induces the lateral cross-linking and conversion of the terminal nitro group to a terminal amino group^32^ and therewith formation of NH_2_-CNMs.

### Azide functionalization of NH_2_-CNM

To functionalize the amino-terminated CNMs, the azidoacetyl chloride linker (2-AAC, 97%, 30% solution in diethyl ether) was grafted to their terminal amino groups^33^.

### Preparation of samples

Antibody solutions (targeting SARS-CoV-2 N-protein and S-protein) were prepared in Immobilization buffer (Cytiva GmbH) at a concentration of 50 µg/mL. A casein solution was prepared in physiological PBS-P buffer (Cytiva GmbH) at a concentration of 100 mM.

Spiked buffer samples were prepared by adding SARS-CoV-2 N-protein and S-protein to the PBS-P buffer of physiological ionic strength. The physiological PBS-P buffer solution was prepared by diluting 10x PBS-P buffer from Cytiva GmbH in ultrapure water in a ratio of 1:10.

Spiked nasopharyngeal swab samples were prepared by adding SARS-CoV-2 S-protein to simulated samples with a complex matrix. To create this matrix, a nasopharyngeal swab was added to a lysate buffer solution provided by the Forschungszentrum für Medizintechnik und Biotechnologie (fzmb GmbH). This lysate buffer closely resembles the buffers commonly used for clinical sample collection.

### SPR measurements and analysis

Surface plasmon resonance investigations were conducted using a commercial multiparametric SPR system (MP-SPR Navi 210 A VASA, Bionavis Ltd., Tampere, Finland) equipped with lasers emitting at three distinct wavelengths (λ = 670 nm, 785 nm, 980 nm). The evaluation of the equilibrium disassociation constant involved analyzing real-time sensorgrams acquired at 670 nm. To determine layer thickness, MP-SPR peak spectral analysis was utilized employing the three wavelengths for fitting and iteration. All assessments were executed through continuous full range angular scanning (40 − 78°) on the three laser wavelengths and in two flow channels with a consistent buffer flow (Physiological buffer, 10 µL/min) and maintaining the temperature at 25 °C. The SPR sensor chips were purchased from Bionavis Ltd. (Tampere, Finland); these sensors consist of glass coated with approximately 50 nm of gold along with a 10 nm chromium adhesion layer.

### X-ray photoelectron spectroscopy

X-ray Photoelectron spectroscopy was performed using an ultra-high vacuum (UHV) Multiprobe system (Scienta Omicron) with a monochromatic X-ray source (Al K_α_) and an electron analyzer (Argus CU) with a resolution of 0.6 eV. For some spectra (antibody-CNM functionalized with casein and N-protein immobilization), a Thermo Scientific KAlpha spectrometer equipped with an Al Kα anode as an X-ray source was used.

### Polarization-modulation infrared reflection absorption spectroscopy

Polarization-modulation infrared reflection absorption spectra (PM-IRRAS) were recorded at a resolution of 4 cm^–1^ using a Fourier transform infrared (FTIR) spectrometer (INVENIO R, Bruker) coupled with a polarization-modulation accessory (PMA50, Bruker). The samples were probed in reflection mode on native Au substrates at a grazing incidence angle of 81.5°. The spectrometer was purged with dry nitrogen at a flow rate of 3 L×min^–1^, and the MCT detector was cooled with liquid nitrogen.

## Supplementary Information

Below is the link to the electronic supplementary material.

Supplementary Material 1

## Data Availability

The data that support the findings of this study are available from the corresponding author upon reasonable request.
