# Supplementary material for: Highly sensitive and label-free detection of SARS-CoV-2 proteins via surface plasmon resonance using biofunctionalization with 1 nm thick carbon nanomembranes
Source: Sci Rep. 2025 Aug 25;15:31248. doi: 10.1038/s41598-025-16342-5 (PMC12378216; doi:10.1038/s41598-025-16342-5)
Supplement: Supplementary file 1 — Supplementary Material 1 [file 41598_2025_16342_MOESM1_ESM.pdf]

Supporting Information

Highly sensitive and label-free detection of SARS-CoV-2 proteins *via* surface plasmon resonance using biofunctionalization with 1 nm thick carbon nanomembranes

Ghazaleh Eshaghi[1], David Kaiser[1], Hamid Reza Rasouli[1], Rania Ennaciri[1], Martha Frey[1], Christof Neumann[1], Dominik Gary[2], Tobias Fischer[2], Katrin Frankenfeld[2], Andrey Turchanin[1,3,4]*

[1]Institute of Physical Chemistry, Friedrich Schiller University Jena, 07743 Jena, Germany
[2]fzmb GmbH, Forschungszentrum für Medizintechnik und Biotechnologie,
99947 Bad Langensalza, Germany
[3]Abbe Center of Photonics, Friedrich Schiller University Jena, 07745 Jena, Germany
[4]Jena Center for Soft Matter (JCSM), 07743 Jena, Germany

[*]Corresponding Author: Andrey Turchanin (andrey.turchanin@uni-jena.de)

1. Methods

1.1. SPR measurements and analysis

Spectral reflectance responses were measured in the Kretschmann configuration[1] using a multiparametric SPR instrument from Bionavis Ltd. (Navi 210A). Angular spectra were recorded simultaneously at 670 nm, 785 nm and 980 nm. The angle for surface plasmon resonance, $\Delta\theta_{SPR}$, was calculated using the software MP-SPR Navi 6.7.0.9 from Bionavis Ltd. NH_2-CNM were synthesized on Au sensor slides from Bionavis Ltd. and further chemically modified to with azide linker molecules as described in the experimental section 4.3 in the main paper. The signal channel of the flow cell was functionalized with 50 µg/ml DBCO-labeled monoclonal SARS-CoV-2 antibodies in 10 mM sodium acetate buffer delivered at a flow rate of 5 µl/min for 25 minutes. The DCBO-labeled antibodies are covalently attached to the azide-terminated CNM (N_3-CNM) *via* click chemistry. The second channel, serving as a reference, was not functionalized with antibodies. After achieving a stable baseline, a 100 µM casein solution in physiological PBS-P buffer was introduced for 10 minutes at a flow rate of 10 µl/min in both channels. Subsequently, varying concentrations of the N-protein or S-protein were injected at a flow rate of 10 µl/min in both channels for 5 minutes and 15 minutes, respectively. The samples with the smallest protein levels were injected first. For S-protein detection, the surface was regenerated after a dissociation time of 10 minutes in physiological PBS-P solution. The regeneration was performed with 10 mM glycine-HCl, pH 2 for 30 seconds at a flow rate of 10 µl/min. For the detection of N-protein, the surface was not regenerated. Several regeneration strategies were also explored for the N-protein, but none of them resulted in removal of the target proteins from the antibody without impairing the antibody's ability to bind to the target specifically. Physiological PBS-P buffer was used as the running buffer throughout all experiments, maintaining a consistent flow rate of 10 µl/min. k_a, k_d and K_D values were calculated using TraceDrawer™ (Langmuir 1:1 binding model) and OriginLab™ 2021b[2,3]. All kinetic

analyses were based on differential sensorgrams, which exhibited stable baselines despite minor drifts in the raw signal and reference channels. This ensured consistent and reproducible binding profiles across concentrations. The limit of detection (*LOD*) for the biosensors defined as the concentration of the analyte that produces a binding response that is three times higher than the noise of the measurement, σ, was determined by comparing the binding response of the samples with the signal of a pure buffer solution[4]. For thickness determination, we employed a dielectric multilayer model (BioNavis LayerSolver™ software version 1.4.0.3). The fitting process involves modeling the optical system with multiple layers, considering the thickness and refractive index of each layer individually. Numerical calculations are iteratively carried out utilizing Fresnel's equations and a transfer matrix formalism.

1.2. X-ray photoelectron spectroscopy (XPS)

XPS was performed using a UHV multiprobe system (Scienta Omicron) with a monochromatic X-ray source (Al K_α) and an electron analyzer (Argus CU) with a resolution of 0.6 eV. The samples antibody-CNM with casein and N-protein-CNM with casein were measured using a Thermo Scientific KAlpha spectrometer equipped with an Al K_α anode as an X-ray source. In order to compare the intensity of all spectra, an Ar^+ sputter-cleaned gold substrate was measured for calibration in both systems and the intensities of all peaks are calibrated using the intensity of the Au $4f_{7/2}$ peaks. For all presented results, the background was subtracted, and spectra were calibrated using the Au $4f_{7/2}$ (84.0 eV) before undergoing fitting using Voigt functions (30:70). The effective thicknesses of the different CNMs were calculated by comparing their attenuated Au $4f_{7/2}$ signal to the signal of a freshly cleaned gold reference sample. Here, Beer-Lambert law for attenuation of the substrate signal was applied using an inelastic mean free path of λ_{IMFP} = 36 Å.

2. X-ray photoelectron spectroscopy analysis

A stepwise XPS characterization of each preparation and functionalization step was performed. In Figure 2a in the main paper, the XP spectra of the NBPT SAM are presented. The N 1s spectrum confirms the presence of the nitro groups due to the peak at a binding energy (BE) of 405.5 eV (purple). The NO_2 group is also visible in the O 1s spectrum. The successful SAM formation is proven by the S 2p spectrum, showing mainly thiolate bonds at a BE of 162.0 eV (light green). In the C 1s spectrum, C-C bonds (red) are visible at a BE of 284.2 eV, accompanied by a shoulder at 285.2 eV (green) assigned to C-S and C-N bonds. After low-energy electron-induced cross-linking into an NH_2-CNM (Figure 2b), the nitro group is reduced to an amino group at 399.2 eV (magenta). In parallel, the O 1s signal is reduced in intensity as only adsorbed hydrocarbons contribute to this spectrum. In the S 2p signal, three signals are visible. Besides the previously detected thiolate bonds, 60 % of the sulfur is found at a BE of 163.2 eV assigned to -S-S- or -SH bonds (dark green) and a new species at a BE of 161.2 eV (dark yellow) according to Au-S bonds arise. These species were formed due to the electron-irradiation-induced cleavage of bonds in the NBPT SAM. The same effect leads to the broadening of the C 1s signal from 1.4 eV (SAM) to 1.5 eV (CNM). The effective thickness remains similar (see Table S1). The results confirm previously published cross-linking of NBPT SAMs[5,6]. After azide functionalization, the signature of the central nitrogen bonds is visible in the N 1s spectrum (Figure 2c, see main paper)[7]. In addition, in the C 1s spectrum, the signal at 289.6 eV (light blue) assigned to N-C=O bonds increase from 3 % to 6 % due to the presence of the azide linker. The effective thickness of the N_3-CNM increases slightly to 14 ± 2 Å. After functionalization with the antibody, the intensity of C 1s, N 1s, and O 1s spectra increase significantly, leading to a clear increase in the effective thickness to 35 ± 5 Å (Figure 2d, Table S1). In contrast, the intensity of the S 2p signal is reduced as sulfur is only present in the binding group to the Au substrate attenuated by the CNM and the bonded functionalization layer. In the C 1s spectrum, the signal assigned to C-N

or sp^3 C-C bonds becomes the most intense (green). Furthermore, the relative intensity of the C-O/C=O bonds (dark blue) and COOH/N-C=O bonds (light blue) increases. The signal assigned to sp^2 C-C and C-H bonds is significantly attenuated as these bonds are preferentially present in the CNM below the functionalization. Consistently with the C 1s spectrum, two strong signals are also visible in the O 1s spectrum, which is assigned to C=O (dark blue) and C-O/C-OH bonds (light blue). For the final two preparation steps (Figure 2e, functionalization with casein, Figure 2f, additional functionalization with N-protein), we found similar chemical species as for the antibody-CNM. Besides the increase in the effective thickness discussed in the main paper, we see a relative increase in C=O and C-N bonds. Therefore, the amount of nitrogen is growing from 13.6 at% (antibody-CNM) to 14.9 at% (casein-CNM) and 16.0 at% (N-protein-CNM), respectively.

Table S1. The thickness of the organic layers on the SPR sensors was obtained by XPS and SPR.

Sample	XPS thickness (nm)	SPR thickness (nm)
NBPT SAM	1.2 ± 0.2	1.2 ± 0.1
NH_2-CNM	1.3 ± 0.2	1.2 ± 0.2
N_3-CNM	1.4 ± 0.2	1.5 ± 0.1
Antibody-CNM	3.5 ± 0.5	5.4 ± 0.5
Antibody-CNM with casein	4.9 ± 0.7	5.8 ± 0.2
Antibody-CNM with casein and N-protein	5.7 ± 0.8	6.8 ± 0.2

3. Multiparametric SPR thickness and refractive index calculation

The LayerSolver™ software from BioNavis enables calculation of the thickness and refractive index of each layer based on the Fresnel equations, which model the reflection and transmission of light at material interfaces. For the glass and metal layers as well as for the buffer solution the standard fitting method was applied. This method calculates both the real and imaginary parts of the refractive index as free parameters to obtain the best-fitting curve from the experimental data. For the N_3-CNM and the biomolecule layers, convergent solutions could not be achieved using the standard method, and thus a simpler fitting method was employed, where imaginary part of the refractive index is set to 0 neglecting the materials absorption. A linear dispersion relation was assumed for the wavelength-dependent real part of the refractive index using the equation $n(\lambda) = n_{785\mathrm{nm}} + \left(\frac{\lambda\,[\mu\mathrm{m}] - 0.785\,\mu\mathrm{m}}{\mu\mathrm{m}}\right) \times \alpha$, where $n_{785\mathrm{nm}}$ is the refractive index at 785 nm, and α is the dispersion coefficient.[8]

We applied the model to 16 different multiparametric SPR curves, 8 curves from one measurement series with the N-protein antibody and 8 curves from one measurement series with the S-protein antibody. From each of the 8 curves, 4 were recorded from the signal channel, and 4 were recorded from the reference channel. From the two experiments, we have chosen an SPR curve recorded with a sensor surface consisting of only N_3-CNM on gold, N-protein or S-protein antibody-CNM on gold in the signal channel, and N_3-CNM in the reference channel, after injection of casein, and after binding of the N-protein or S-protein, respectively.

Tables S2 and S3, corresponding to the detection of N-protein and S-protein respectively, present the optical constants for Au and Cr used in the calculations along with the fitted parameters for layer thickness and refractive indices at three distinct wavelengths for the respective metal layers. Initially, for the fitting of the N_3-CNM spectra, the thicknesses and refractive indices of the Cr, Au, and N_3-CNM layers were all treated as variables. For subsequent functionalization steps, the thickness and refractive indices of the underlying layers were held

constant, while the parameters for each new layer were recalculated. This consistent approach was employed for all functionalization steps. The model calculations in Figure 2c are in good agreement with the experimental data yielding the values of χ^2 between $0.01 < \chi^2 < 0.3$, where $\chi^2 = \sum \frac{\left(S(\theta)_i - S(\theta)_{sim,i}\right)^2}{S(\theta)_{sim,i}}$ is the sum of the squared differences between observed and simulated reflectance values $S(\theta)_i$ and $S(\theta)_{sim,i}$. We applied the model to describe the experimental data in the range of θ=58°-78°.

Table S2. Results for layer thickness, h_{eff}, real part of refractive index, n, extinction coefficient, κ, and dispersion coefficient, α, obtained from the SPR data upon functionalization of the surface for N-protein detection. Data were analyzed using LayerSolver™ software at three distinct wavelengths (670 nm, 785 nm and 980 nm) for both signal and reference channel. The values for the χ^2-goodness of the model are presented.

λ(nm)	h_{eff} *(nm)*		n		κ		χ^2	
	Signal	Reference	Signal	Reference	Signal	Reference	Signal	Reference
Glass								
670	0		1.5020	1.52020			0.13	0.25
785			1.5162	1.51620	0		0.02	0.14
980			1.5129	1.50780			0.05	0.04
Cr								
670			3.90770	3.44209	4.17821	4.44287	0.13	0.25
785	2.17	1.8	4.07942	3.72045	4.25558	4.43755	0.02	0.14
980			4.22407	4.60589	4.80766	4.41779	0.05	0.04
Au								
670			0.21647	0.20551	3.90760	3.90214	0.13	0.25
785	50.84	52.30	0.22490	0.21621	4.96171	4.95098	0.02	0.14
980			0.25642	0.24690	6.42932	6.44185	0.05	0.04

λ(nm)	h_{eff} *(nm)*		n		α		χ^2	
	Signal	Reference	Signal	Reference	Signal	Reference	Signal	Reference
N_3-CNM								
785	1.55	1.61	1.58934	1.60022	-0.02135	-0.02324	0.23<br>0.09<br>0.12	0.29<br>0.02<br>0.15
Antibody								
785	3.89	-	1.38728	-	-0.00382	-	0.25<br>0.14<br>0.06	-<br>-<br>-
Antibody + Casein								
785	4.30	0.76	1.39763	1.40677	-0.00416	-0.08202	0.27<br>0.13<br>0.06	0.13<br>0.02<br>0.05
Antibody + Casein + N-protein								
785	5.29	1.12	1.41909	1.41711	-0.00495	-0.07131	0.24<br>0.02<br>0.07	0.26<br>0.01<br>0.08

λ(nm)	h_{eff} *(nm)*		n		κ		χ^2	
	Signal	Reference	Signal	Reference	Signal	Reference	Signal	Reference
PBS-P buffer								
670			1.32200	1.33340			0.13	0.25
785	0		1.33340	1.33050	0		0.02	0.14
980			1.33050	1.32200			0.05	0.04

Table S3. Results for layer thickness, h_{eff}, real part of refractive index, n, extinction coefficient, κ, and dispersion coefficient, α, obtained from the SPR data upon functionalization of the surface for S-protein detection. Data were analyzed using LayerSolver™ software at three distinct wavelengths (670 nm, 785 nm and 980 nm) for both signal and reference channel. The values for the χ^2-goodness of the model are presented.

λ(nm)	***h_{eff} (nm)***		***n***		***κ***		***χ^2***	
	**Signal**	**Reference**	**Signal**	**Reference**	**Signal**	**Reference**	**Signal**	**Reference**
Glass								
670	0		1.5020	1.52020			0.18	0.25
785			1.51490	1.51490	0		0.08	0.02
980			1.5129	1.50780			0.29	0.15
Cr								
670			3.60175	3.32956	4.81731	4.53865	0.18	025
785	0.89	1.40	3.75761	3.78146	4.09924	4.84250	0.08	0.02
980			4.31681	4.15590	4.00496	4.71141	0.25	0.15
Au								
670			0.20111	0.20384	3.79844	6.01686	0.18	025
785	54.77	52.16	0.25279	0.26270	4.19196	4.22450	0.08	0.02
980			0.28939	0.27974	6.41978	4.54035	0.28	0.15

λ(nm)	***h_{eff} (nm)***		***n***		***α***		***χ^2***	
	**Signal**	**Reference**	**Signal**	**Reference**	**Signal**	**Reference**	**Signal**	**Reference**
N_3-CNM								
785	1.36	1.40	1.59918	1.60142	-0.02213	-0.02142	0.15<br>0.05<br>0.28	0.29<br>0.02<br>0.21
Antibody								
785	3.98	-	1.40233	-	-0.00425	-	0.20<br>0.07<br>0.15	-<br>-<br>-
Antibody + Casein								
785	4.42	0.88	1.41763	1.40177	-0.00312	-0.09232	0.23<br>0.04<br>0.16	0.22<br>0.02<br>0.24
Antibody + Casein + S-protein								
785	5.35	0.87	1.41536	1.39911	-0.00415	-0.01132	0.17<br>0.03<br>0.07	0.21<br>0.01<br>0.08

λ(nm)	***h_{eff} (nm)***		***n***		***κ***		***χ^2***	
	**Signal**	**Reference**	**Signal**	**Reference**	**Signal**	**Reference**	**Signal**	**Reference**
PBS-P buffer								
670			1.32800	1.33150			0.27	0.29
785	0		1.33580	1.33840	0		0.04	0.03
980			1.33080	1.33020			0.18	0.16

4. Binding of S-protein antibody and casein passivation

Figure S1a shows the obtained sensorgram for signal and reference channel. For the functionalization of the surface with antibodies, the antibody solution in acetate buffer is injected into the signal channel, while pure acetate buffer is injected in the reference channel. Figure S1b shows the differential sensorgram ($\Delta\theta_{SPR}^{*}$) for S-protein antibody, in which the bulk effect is eliminated by subtraction of $\Delta\theta_{SPR}$ of the reference from $\Delta\theta_{SPR}$ of the signal channel. Figure S1c shows the sensorgram of casein immobilization on both signal and reference channels. The presence of the already-immobilized antibody on the signal channel reduces the binding of casein molecules.

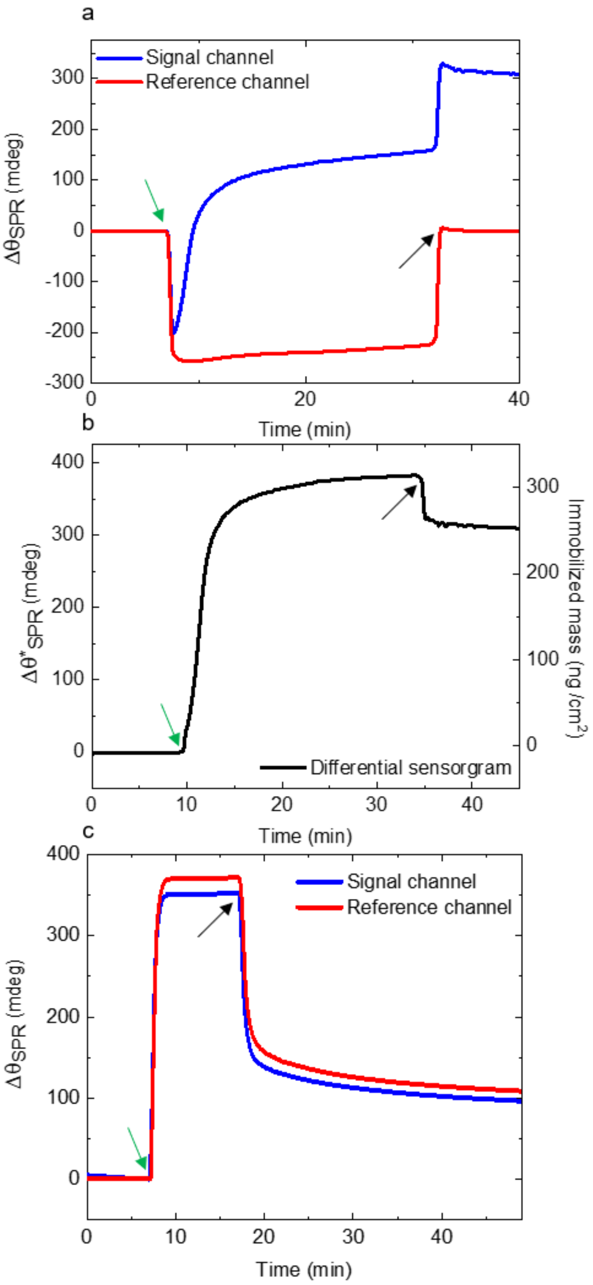

Figure S1. Surface biofunctionalization of N_3-CNM with SARS-CoV-2 S-protein antibody and casein molecules. (a) Real-time resonance angle shift ($\Delta\theta_{SPR}$) during the S-protein antibody immobilization for the signal (blue curve) and the reference (red curve) channels (b) Differential sensorgram ($\Delta\theta^*_{SPR}$) during the S-protein antibody immobilization. (c) Real-time $\Delta\theta_{SPR}$ changes during the surface passivation with casein for the signal (blue curve) and the reference (red curve) channels. The start and the end of the injections are shown with green and black arrows, respectively.

5. Calculation of the surface coverage

We compare the surface coverage values calculated from measurements of the differential binding response, $\Delta\theta^{*}_{SPR}$, at 670 nm, ϕ_1, and the solution of the Fresnel equations for the SPR curves measured at 670 nm, 785 nm, and 980 nm, ϕ_2. To calculate surface coverage from the $\Delta\theta^{*}_{SPR}$ measurements, we first compare the thickness of the functional layer in our experiments with the decay length, l_d, of the system. The decay length refers to the characteristic distance at which the evanescent field that induces surface plasmon polaritons decays to 1/e. The maximum thickness of the functional layer consisting of ~1.5 nm N_3-CNM, ~13 nm antibody[9] and ~4 nm target proteins can be estimated as 20 nm. It has been reported in the literature[10] that at 670 nm in 0.1 M KNO_3, $l_d = 283\ nm$. As we similarly performed that experiments at 670 nm and in a similar buffer (0.17 M PBS-P), the functional layer is much thinner than the decay length in our experiments. Under these conditions, the surface mass density, Γ, can be approximated as[11] $\Gamma = \frac{l_d}{2}\frac{\Delta\theta_{SPR}}{\tilde{m}\frac{dn}{dc}}$, where $\tilde{m}$ is the specific sensitivity, $\frac{dn}{dc} = (n_p - n_s)\vartheta_p$ is the refractive index increment, c is the biomolecule concentration, n_p is the refractive index of the biomolecule, n_s is the refractive index of the solvent, and ϑ_p is the partial specific volume of the biomolecule. For our setup, $\frac{l_d}{2}\left(\tilde{m}\frac{dn}{dc}\right)^{-1} = 0.82\frac{ng}{mdeg\ cm^2}$ at a wavelength of 670 nm for typical biomolecules.[8]

At the point of saturation in the kinetic binding assay, the differential binding response $\Delta\theta^{*}_{SPR}$ represents the maximum surface coverage. The saturation level was clearly visible in the binding kinetics when antibodies were immobilized. For the target N- and S-proteins, we calculate the surface coverage for a reporting point at the end of the injection of the last sample of the concentration series. Given the surface mass density, Γ, and the known molecular mass of the biomolecule, M, the number of molecules per unit area, N, is given by $N = \frac{\Gamma}{M}$. The surface

coverage can be calculated as $\phi_1 = \frac{N}{N_{\max}}$, where $N_{\max}$ is the maximum possible surface number density of biomolecules.

To estimate $N_{\max}$ for the antibody immobilization by covalent binding to the N_3-CNM considering that due to the covalent binding of the DBCO label to the N_3-CNM, the formation of a multilayer is not likely. The thickness of a closed monolayer of antibodies is estimated to be equal to the effective molecular diameter d of the antibodies. Considering hexagonal close packing as the most densely packed structure, the theoretical maximum surface number density $N_{\max}$ of the antibodies can be estimated by $N_{\max} = \frac{2}{\sqrt{3} \cdot d^2}$. With the effective molecular diameter of IgG antibodies,[12] $d = 5.5 \pm 0.5\ nm$, $N_{\max} = 3.8\ \pm\ 0.4\ \times 10^{12}\ cm^{-2}$. As the antibodies that target the SARS-CoV-2 S- and N-proteins possess two paratopes for specific capturing, the largest possible surface number density of proteins due to specific binding is given by $N_{\max}^{prot} = 2N_{Ab}$, where N_{Ab} is the density of the surface number density of the antibodies on the surface.

The second method for calculating surface coverage involves determining the effective thickness increase of the adsorbed biomolecular layer using multiparametric SPR. In this approach, the effective thickness, h_{eff}, is derived by solving the Fresnel equations for the SPR curve at three wavelengths: 670 nm, 785 nm and 980 nm. The surface coverage, ϕ_2, can then be calculated as the ratio of the effective thickness of the biomolecule layer to the height h of one individual biomolecule, $\phi_2 = \frac{h_{eff}}{h}$. For consider that the thickness of a closed film may correspond to the effective hydrodynamic diameter of the biomolecules. For IgG antibodies, $h_{IgG} = 12.7 \pm 3.0\ nm$.[9] Assuming similar density for the S-protein RBD and the N-protein, and considering their difference in molecular mass M, we estimate $h_{NP} = \sqrt[3]{\frac{M_{NP}}{M_{IgG}}} = 8 \pm 2\ nm$ and $h_{SP} = 7 \pm 2\ nm$.

Table S4 shows the results obtained for ϕ_1 and ϕ_2. In order to calculate a reliable value for the surface coverage, we average ϕ_1 and ϕ_2. This approach integrates findings from two complementary methodologies, one focusing on molecular aspects and the other on structural considerations. By averaging results from these methods, we reduce the influence of method-specific biases, as uncertainties unique to each method can potentially offset one another. For the surface coverage of the antibodies, we obtain $\phi = 0.26 \pm 0.07$ for the Ab_{NP} and $\phi = 0.29 \pm 0.08$ for the Ab_{SP}. For the target proteins, considering only specific adsorption, the surface coverage relative to the available binding sites is $\phi_1 = 0.27 \pm 0.12$ for the N-protein and $\phi_1 = 0.35 \pm 0.09$ for the S-protein. Additionally, the surface coverage relative to the maximum surface number density of a monolayer is $\phi_2 = 0.13 \pm 0.06$ for both the N-protein and the S-protein.

Table S4. Surface coverage of the antibodies (Ab_{NP}, Ab_{SP}) and antigens (NP, SP) on the SPR sensor calculated from the measurement of $\Delta\theta^*_{SPR}$ at 670 nm, ϕ_1, and the solution of the Fresnel equations for the SPR curves measured at 670 nm, 785 nm and 980 nm, ϕ_2

Protein	$\Delta\theta^*_{SPR}$ (mdeg)	Γ (ng/ cm^2)	M (kDa)	N (10^{12} cm^{-2})	d (nm)	N_{max} (10^{12} cm^{-2})	ϕ_1 (%)	h_{eff} (nm)	h (nm)	ϕ_2 (%)
Ab_{NP}	250 ± 6	208 ± 5	150	8 ± 1	5 ± 1	38 ± 4	21 ± 5	3.9 ± 0.5	12.7 ± 3.0	31 ± 12
Ab_{SP}	309 ± 7	257 ± 6	150	10 ± 1	5 ± 1	38 ± 4	26 ± 5	4.0 ± 0.5	12.7 ± 3.0	32 ± 12
NP	27 ± 2	23 ± 2	46	3 ± 1		16 ± 2	27 ± 12	1.0 ± 0.2	8 ± 2	13 ± 6
SP	44 ± 3	38 ± 3	32	7 ± 1		20 ± 2	35 ± 9	0.9 ± 0.2	7 ± 2	13 ± 6

6. Surface passivation of the N-protein antibody-functionalized N_3-CNM

In order to decrease non-specific adsorption, we injected protein-repellent agents after the functionalization step of the N_3-CNM with antibodies. Figure S2 shows the sensorgrams of both signal and reference channels during the immobilization of the dibenzocyclooctyne labeled polyethylene glycol (DBCO-PEG) 20kDa, the DBCO-PEG 5kDa, and sequential DBCO-PEG 5kDa and BSA on the N-protein antibody-functionalized N_3-CNM. The binding response of the blocking agents on the reference channel is 52 ± 13 mdeg, 22 ± 8 mdeg, and 30 ± 7 mdeg for polyethylene glycol (DBCO-PEG) 20kDa, the DBCO-PEG 5kDa, and sequential DBCO-PEG 5kDa and BSA, compared to the lower responses observed in the signal channels of 33 ± 9 mdeg, 11 ± 5 mdeg, and 20 ± 6 mdeg, respectively. The difference in binding response between the signal and reference channels can be attributed to the presence of antibodies solely on the signal channel.

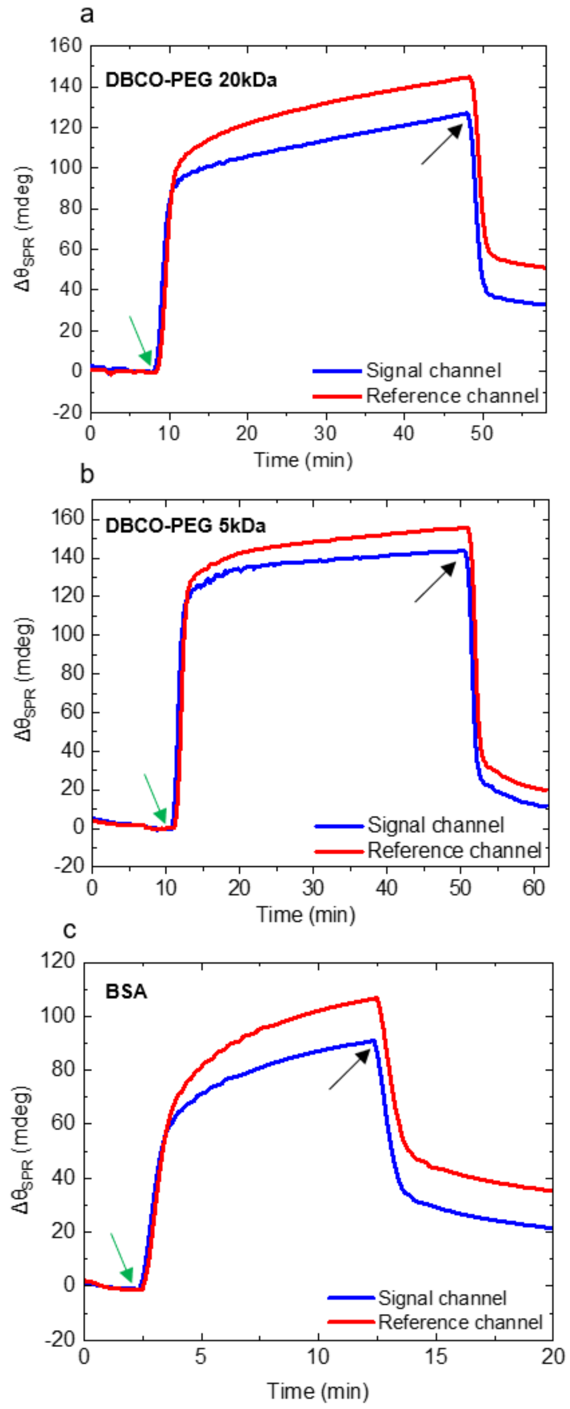

Figure S2. Surface passivation of the N-protein antibody-functionalized N_3-CNM with different blocking agents in the SPR flow cell. Real-time resonance angle shift ($\Delta\theta_{SPR}$) sensorgrams during the functionalization with (a) DBCO-PEG 20kDa, (b) DBCO-PEG 5kDa, and (c) BSA after DBCO-PEG 5kDa. Blue curve: signal channel, red curve: reference channel. The injections' start and end are shown with green and black arrows, respectively.

7. Effect of the blocking agents on the N-protein detection

Samples treated with different blocking agents were analyzed for N-protein detection. Figures S3a, S3c and S3e show sensorgrams of the signal and the reference channel during an injection of a series of N-protein containing samples. The limit of detections (*LOD*s) after PEG 20kDa, PEG 5kDa, and sequential PEG 5kDa and BSA passivation were 1.13 nM, 0.73 nM, and 0.51 nM, respectively, much larger than the *LOD* obtained with casein passivation. We attribute the high signal to noise ratio after passivation with casein to the large molecular weight and more heterogeneous molecular structure of casein resulting in more efficient blocking of non-specific adsorption. The corresponding differential sensorgrams ($\Delta\theta^{*}_{SPR}$) for each blocking agent are shown in Figures S3b, S3d, and S3f, respectively, together with single-cycle kinetic model fittings. The k_a, k_d, K_D and *LOD* values obtained based on the kinetic analysis for the N-protein interaction are presented in Table S5.

Table S5. Results of the kinetic analysis of the SARS-CoV-2 N-protein binding on the antibody functionalized N_3-CNM using different blocking agents.

Blocking agents	k_a ($M^{-1}s^{-1}$)	k_d (s^{-1})	K_D (nM)	~3σ (mdeg)	~LOD (nM)
PEG 20kDa	$(50 \pm 4) \times 10^{4}$	$(149 \pm 10) \times 10^{-5}$	2.98 ± 0.28	0.75	1.13
PEG 5kDa	$(87 \pm 6) \times 10^{4}$	$(149 \pm 10) \times 10^{-5}$	1.71 ± 0.17	0.75	0.75
PEG 5kDa + BSA	$(69 \pm 5) \times 10^{4}$	$(85 \pm 6) \times 10^{-5}$	1.23 ± 0.12	0.75	0.51
Casein	$(300 \pm 18) \times 10^{4}$	$(170 \pm 10) \times 10^{-5}$	0.57 ± 0.05	0.60	0.19

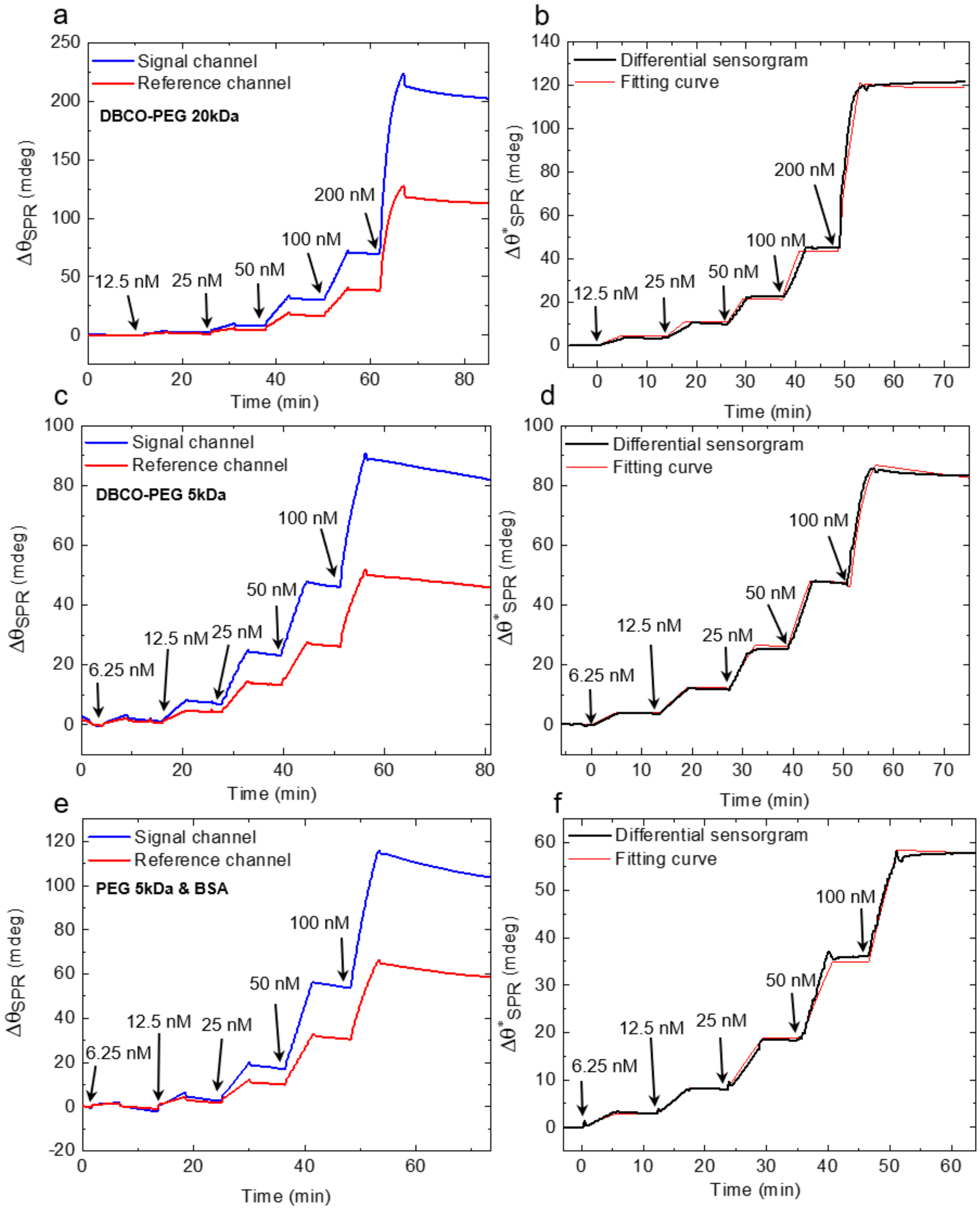

Figure S3. Real-time shift of the $\Delta\theta_{SPR}$ during parallel injection of PBS-P buffer solution spiked with N-protein into signal (blue curve) and reference (red curve) channel after surface passivation with (a) DBCO-PEG 20kDa, (c) DBCO-PEG 5kDa and (e) BSA after DBCO-PEG 5kDa. (b,d,f) Corresponding differential sensorgrams ($\Delta\theta^{*}_{SPR}$) (black curve) and single cycle kinetics model fits.

8. Regeneration of N-protein

Figures S4a and S4b show that less than 10% of the bound antigens were removed after a single regeneration cycle.

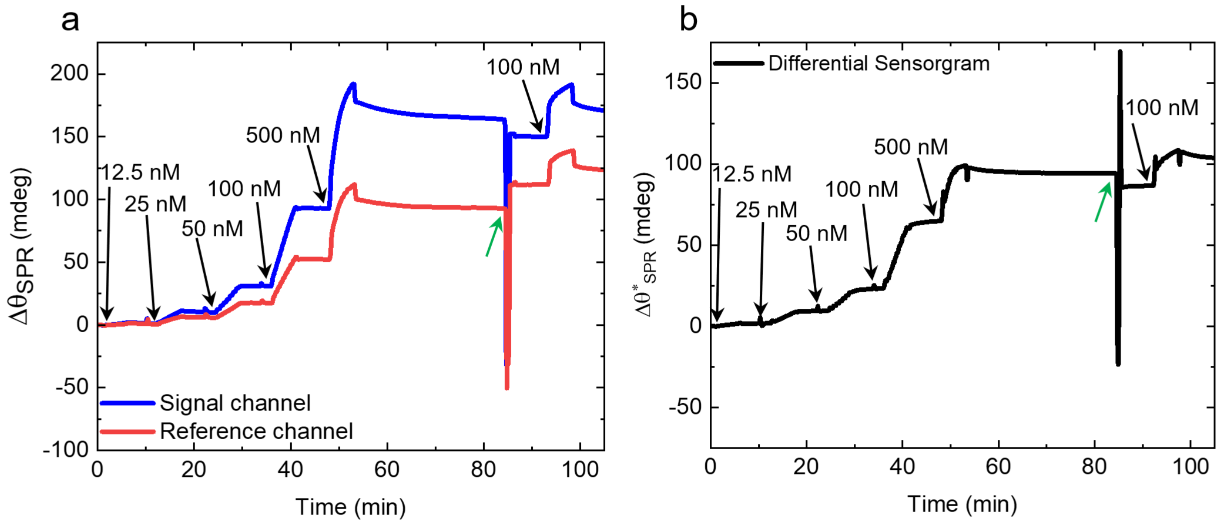

Figure S4. SPR-based detection of SARS-CoV-2 N-protein using antibody-functionalized N_3-CNM. (a) Real-time resonance angle shift ($\Delta\theta_{SPR}$) during the series of N-protein concentration injections (5 minutes for each concentration) into the signal (blue curve) and the reference (red curve) channels. The start of the injections and regeneration are shown with balck and green arrows, respectively. (b) Differential sensorgrams $\Delta\theta^{*}_{SPR}$during the series of N-protein concentration injections.

9. Differential sensorgrams of N-protein and S-protein detection at different concentrations

Figures S5a and S4b display the differential sensorgrams for the N-protein concentration series, illustrating the responses at both low and high concentration ranges. Figure S5c presents the differential sensorgram corresponding to the S-protein at low concentrations.

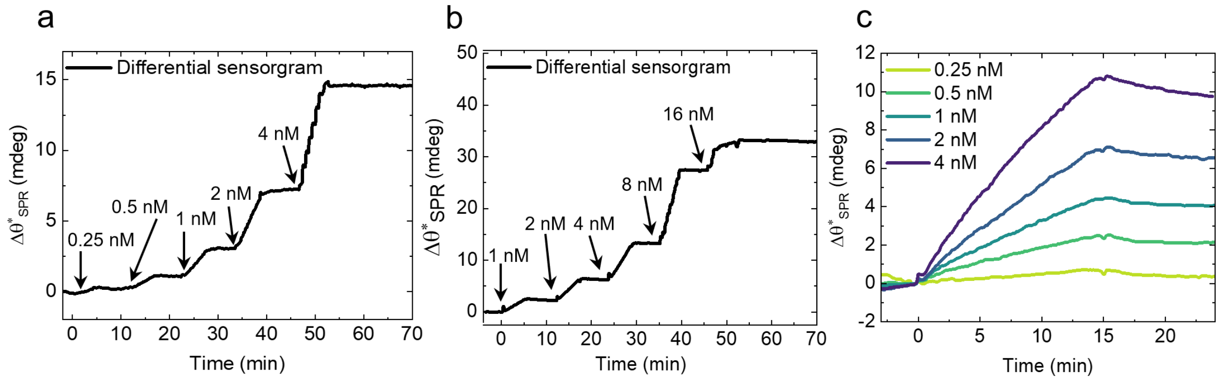

Figure S5. SPR-based detection of SARS-CoV-2 N-protein and S-protein using antibody-functionalized N_3-CNM. (a, b) Differential sensorgram ($\Delta\theta_{SPR}^{*}$) during the series of N-protein concentration injections. The start of the injections is shown with black arrows. (c) Differential sensorgram $\Delta\theta_{SPR}^{*}$during the series of S-protein concentration injections.

10. S-protein detection

The S-protein detection in undiluted physiological PBS-P buffer and nasopharyngeal swab sample is investigated after the surface passivation functionalization with casein. The corresponding differential sensorgrams (multi-cycle kinetic) were fitted by Langmuir 1:1 binding model. The k_a, k_d, K_D, R_{max} and LOD values obtained based on the kinetic analysis for the S protein interaction are presented in Tables S6,7.

Table S6. Results of the kinetic analysis of the SARS-CoV-2 S-protein binding on the antibody functionalized N_3-CNM in physiological PBS-P buffer.

c (nM)	R_{max} *(mdeg)*	k_a $(M^{-1}s^{-1})$	k_d (s^{-1})	K_D *(pM)*	*~3σ (mdeg)*	*~LOD (pM)*
0.1	57 ± 4	(200 ± 14) $\times 10^4$	(4.5 ± 0.3) × 10^{-5}	22 ± 2	0.75	10
0.2	59 ± 4					
0.4	54 ± 4					
0.8	47 ± 3					
1.6	45 ± 3					

Table S7. Results of the kinetic analysis of the SARS-CoV-2 S-protein binding on the antibody functionalized N_3-CNM in undiluted nasopharyngeal swab samples

c (nM)	R_{max} *(mdeg)*	k_a $(M^{-1}s^{-1})$	k_d (s^{-1})	K_D *(pM)*	*~3σ (mdeg)*	*~LOD (pM)*
0.1	49 ± 3	(52 ± 4) $\times 10^4$	(5.7 ± 0.4) × 10^{-5}	109 ± 10	2.1	40
0.2	52 ± 4					
0.4	47 ± 3					
0.8	40 ± 3					
1.6	37 ± 3					

11. Calculation of the clinically relevant range for the SARS-CoV-2 S-protein in rapid antigen test solutions

In order to estimate the clinically relevant range of the SARS-CoV-2 N- and S-proteins for the diagnosis of SARS-CoV-2 in rapid antigen test solutions we first compared different literature reports on virus loads measured in frame of the clinical diagnosis in patients infected with COVID-19. $10^{7.5}$-$10^{9.5}$ viral RNA copies/ml were found by Olha Puhach et al.[13] in patients infected with the Omicron and Delta variants. For the Delta variant, Rebecca Earnest et al.[14] reported an average of $10^{7.3}$ viral RNA copies/ml. Note that, when collecting nasopharyngeal swab specimens for RT-PCR experiments, they are typically extracted in 1-3 ml transport media[15,16]. Instead, for rapid antigen detection, only 0.3 - 1 ml of extraction solution is typically used[17]. Using different extraction volumes can significantly affect the viral load, leading to concentration increases of up to 10 times (up to $10^{8.3}$-$10^{10.5}$ viral RNA copies/ml)[15-17]. We therefore correct for the difference in sample volume for RT-PCS and the rapid antigen test. ~50 spike trimmers are reported by Steffen Klein et al. per virion for the post-Wuhan variant[18]. Since each spike monomer has one S1 subunit, there are ~150 S1 proteins per virion. Accordingly, the clinically relevant range for S-protein can be estimated to be between $c_{min}^{SP} = N_A^{-1} 10^{8.3} \frac{virion}{ml} 150 \frac{1}{virion} 10^3 \frac{ml}{l} = 50\ pM$ and $c_{max}^{SP} = 7.8\ nM$.

References

1 Kretschmann, E. & Raether, H. Notizen: Radiative Decay of Non Radiative Surface Plasmons Excited by Light. *Z NATURFORSCH A* **23**, 2135-2136 (1968). https://doi.org:doi:10.1515/zna-1968-1247

2 Goutelle, S. *et al.* The Hill equation: a review of its capabilities in pharmacological modelling. Fundam. Clin. Pharmacol. **22**, 633-648 (2008). https://doi.org/10.1111/j.1472-8206.2008.00633.x

3 Langmuir, I. The adsorption of gases on plane surfaces of glass, mica and platinum. *J. Am. Chem. Soc.* **40**, 1361-1403 (1918). https://doi.org/10.1021/ja02242a004

4 Homola, J. & Piliarik, M. in *Surface Plasmon Resonance Based Sensors* (ed Jiří Homola) 45-67 (Springer Berlin Heidelberg, 2006). https://doi.org/10.1007/5346_029

5 Eck, W. *et al.* Generation of Surface Amino Groups on Aromatic Self-Assembled Monolayers by Low Energy Electron Beams—A First Step Towards Chemical Lithography. *Adv. Mater.* **12**, 805-808 (2000).

6 Neumann, C., Wilhelm, R. A., Küllmer, M. & Turchanin, A. Low-energy electron irradiation induced synthesis of molecular nanosheets: influence of the electron beam energy. *Faraday Discuss.* **227**, 61-79 (2021). https://doi.org/10.1039/D0FD00127A

7 Gouget-Laemmel, A. C. *et al.* Functionalization of Azide-Terminated Silicon Surfaces with Glycans Using Click Chemistry: XPS and FTIR Study. *J. Phys. Chem. C* **117**, 368-375 (2013). https://doi.org:10.1021/jp309866d

8 Marquart, A., Kuncova-Kallio, J., Albers, M., Bombera, R., Stahlberg, R. *Handbook of MP/SPR*. 1st edn, (Bionavis Ltd., 2019). https://www.bionavis.com/handbook

9 List of protein hydrodynamic diameters DH. Dynamic Biosensors GmbH (HQ), 2017).

10 Haemers, S., Koper, G. J., van der Leeden, M. C. & Frens, G. An alternative method to quantify surface plasmon resonance measurements of adsorption on flat surfaces. *Langmuir* **18**, 2069-2074 (2002). https://doi.org/10.1021/la011379g

11 Ball, V. & Ramsden, J. J. Buffer dependence of refractive index increments of protein solutions. *Biopolymers* **46**, 489-492 (1998).

12 Thomas, G. D. in *Drug Targeting: Strategies, Principles, and Applications* (eds G. E. Francis & Cristina Delgado) 115-132 (Humana Press, 2000). https://doi.org/10.1385/1-59259-083-7:115

13 Puhach, O. *et al.* Infectious viral load in unvaccinated and vaccinated patients infected with SARS-CoV-2 WT, Delta and Omicron. *MedRxiv*, 2022.2001. 2010.22269010 (2022). https://doi.org/10.1101/2022.01.10.22269010

14 Earnest, R. *et al.* Comparative transmissibility of SARS-CoV-2 variants delta and alpha in New England, USA. *Cell Rep. Med.***3** (2022). https://doi.org/10.1016/j.xcrm.2022.100583

15 Stower, H. Virological assessment of SARS-CoV-2. *Nat. Med.* **26**, 465-465 (2020). https://doi.org/10.1038/s41591-020-0847-6

16 Zheng, S. *et al.* Viral load dynamics and disease severity in patients infected with SARS-CoV-2 in Zhejiang province, China, January-March 2020: retrospective cohort study. *BMJ* **369**, m1443 (2020). https://doi.org:10.1136/bmj.m1443

17 Piccinini, E. *et al.* Surface Engineering of Graphene through Heterobifunctional Supramolecular-Covalent Scaffolds for Rapid COVID-19 Biomarker Detection. *ACS Appl. Mater. Interfaces.* **13**, 43696-43707 (2021). https://doi.org:10.1021/acsami.1c12142

18 Klein, S. *et al.* SARS-CoV-2 structure and replication characterized by in situ cryo-electron tomography. *Nat. Commun.* **11**, 5885 (2020). https://doi.org/10.1038/s41467-020-19619-7
